# Histone H3 Variant Regulates RNA Polymerase II Transcription Termination and Dual Strand Transcription of siRNA Loci in *Trypanosoma brucei*

**DOI:** 10.1371/journal.pgen.1005758

**Published:** 2016-01-21

**Authors:** David Reynolds, Brigitte T. Hofmeister, Laura Cliffe, Magdy Alabady, T. Nicolai Siegel, Robert J. Schmitz, Robert Sabatini

**Affiliations:** 1 Department of Biochemistry and Molecular Biology, University of Georgia, Athens, Georgia, United States of America; 2 Institute of Bioinformatics, University of Georgia, Athens, Georgia, United States of America; 3 Department of Plant Biology, University of Georgia, Athens, Georgia, United States of America; 4 Research Center for Infectious Diseases, University of Wuerzburg, Wuerzburg, Germany; 5 Department of Genetics, University of Georgia, Athens, Georgia, United States of America; Universidade de Lisboa Instituto de Medicina Molecular, PORTUGAL

## Abstract

Base J, β-D-glucosyl-hydroxymethyluracil, is a chromatin modification of thymine in the nuclear DNA of flagellated protozoa of the order Kinetoplastida. In *Trypanosoma brucei*, J is enriched, along with histone H3 variant (H3.V), at sites involved in RNA Polymerase (RNAP) II termination and telomeric sites involved in regulating variant surface glycoprotein gene (*VSG*) transcription by RNAP I. Reduction of J in *T*. *brucei* indicated a role of J in the regulation of RNAP II termination, where the loss of J at specific sites within polycistronic gene clusters led to read-through transcription and increased expression of downstream genes. We now demonstrate that the loss of H3.V leads to similar defects in RNAP II termination within gene clusters and increased expression of downstream genes. Gene derepression is intensified upon the subsequent loss of J in the *H3*.*V* knockout. mRNA-seq indicates gene derepression includes *VSG* genes within the silent RNAP I transcribed telomeric gene clusters, suggesting an important role for H3.V in telomeric gene repression and antigenic variation. Furthermore, the loss of H3.V at regions of overlapping transcription at the end of convergent gene clusters leads to increased nascent RNA and siRNA production. Our results suggest base J and H3.V can act independently as well as synergistically to regulate transcription termination and expression of coding and non-coding RNAs in *T*. *brucei*, depending on chromatin context (and transcribing polymerase). As such these studies provide the first direct evidence for histone H3.V negatively influencing transcription elongation to promote termination.

## Author Summary

*Trypanosoma brucei* is an early-diverged parasitic protozoan that causes African sleeping sickness in humans. The genome of *T*. *brucei* is organized into polycistronic gene clusters that contain multiple genes that are co-transcribed from a single promoter. Because of this genome arrangement, it is thought that all gene regulation in *T*. *brucei* occurs after transcription at the level of RNA (processing, stability, and translation). We have recently described the presence of a modified DNA base J and variant of histone H3 (H3.V) at transcription termination sites within gene clusters where the loss of base J leads to read-through transcription and the expression of downstream genes. We now find that H3.V also promotes termination prior to the end of gene clusters, thus regulating the transcription of specific genes. Additionally, H3.V inhibits transcription of siRNA producing loci. Our data suggest H3.V and base J are utilized for regulating gene expression via terminating transcription within polycistronic gene arrays and regulating the synthesis of siRNAs in trypanosomes. These findings significantly expand our understanding of epigenetic regulatory mechanisms underlying transcription termination in eukaryotes, including divergent organisms that utilize polycistronic transcription, providing the first example of a histone variant that promotes transcription termination.

## Introduction

Kinetoplastids are early-diverged protozoa that include the human parasites *Trypanosoma brucei*, *Trypanosoma cruzi*, and *Leishmania major*, which cause African sleeping sickness, Chagas disease, and leishmaniasis, respectively. The genomes of kinetoplastids are arranged into long gene clusters, or polycistronic transcription units (PTUs), which are transcribed by RNA polymerase (RNAP) II [1–3]. RNAP II transcription initiation and termination occurs at regions flanking PTUs called divergent strand switch regions (dSSRs) and convergent strand switch regions (cSSRs), respectively [4]. Pre-messenger RNAs (mRNA) are processed to mature mRNA with the addition of a 5’ spliced leader sequence through *trans*-splicing, followed by 3’ polyadenylation [5–10]. The arrangement of genes into PTUs has led to the assumption that transcription is an unregulated process in these eukaryotes and a model in which gene regulation occurs strictly post-transcriptionally [11, 12]. However, specific chromatin marks have been characterized at sites of transcription initiation and termination, including histone variants and modified DNA base J, which could function to regulate polycistronic transcription and gene expression [13–17].

Base J, β-D-glucosyl-hydroxymethyluracil, is a modified DNA base consisting of O-linked glycosylation of thymine in the genome of kinetoplastids and closely related unicellular flagellates [18, 19]. Whilst J is largely a telomeric modification, it is also found internally within chromosomes at RNAP II transcription initiation and termination sites [13, 20–25]. As reviewed in Borst and Sabatini (2008), analysis of RNAP I transcribed telomeric polycistronic units in *T*. *brucei* led to the discovery of base J [20, 26]. Regulation of the ~15 telomeric variant surface glycoprotein expression sites (*VSG* ESs) allows the parasite to evade the host immune system in a process called antigenic variation [27, 28]. Although the genome of *T*. *brucei* has over 1,000 *VSG* genes, only one *VSG* is expressed at a given time. This is achieved through regulated transcription of the telomeric ESs, only one of which is productively transcribed at any time. The association of the modified base with silent ESs in the bloodstream life-cycle stage of the parasite has led to the hypothesis that J plays a role in the regulation of antigenic variation. However, no direct evidence has been provided.

Base J is synthesized in a two-step pathway in which a thymidine hydroxylase, JBP1 or JBP2, hydroxylates thymidine residues at specific positions in DNA to form hydroxymethyluracil, followed by the transfer of glucose to hydroxymethyluracil by the glucosyltransferase, JGT [26, 29, 30]. JBP1 and JBP2 belong to the TET/JBP subfamily of dioxygenases, which require Fe^2+^ and 2-oxoglutarate for activity [31–34]. The synthesis of base J can be inhibited by competitive inhibition of the thymidine hydroxylase domain of JBP1 and JBP2 by dimethyloxalylglycine (DMOG), a structural analog of 2-oxoglutarate [31, 35]. Removal of both JBP1 and JBP2 or the JGT also results in *T*. *brucei* cells devoid of base J [29–31, 36].

The co-localization of base J with modified and variant histones at dSSRs and cSSRs suggested a functional role of modified DNA in the regulation of RNAP II transcription [13]. Our work in *T*. *cruzi* described a unique role of J in regulating RNAP II transcription initiation, where the loss of base J resulted in the formation of more active chromatin, increased RNAP II recruitment and increased PTU transcription rate [24, 37]. Recent studies have described a role for base J regulating RNAP II termination in *T*. *brucei* and *Leishmania*. van Luenen et al. (2012) found that reduction of base J in *L*. *tarentolae* is associated with the generation of RNAs downstream of the cSSR that are antisense to the genes on the opposing gene cluster [25]. Reduction of base J in *L*. *major* resulted in similar defects [35]. Strand-specific RT-PCR detection of the nascent transcript confirmed that the J-dependent generation of RNAs downstream of the cSSR is due to read-through transcription at cSSR termination sites. In contrast, loss of J in *T*. *brucei* failed to indicate any defect in termination at cSSRs [35]. However, we localized base J at sites within PTUs where the loss of J led to read-through transcription and upregulated expression of downstream genes. Therefore, base J is required for RNAP II termination in both *Leishmania* and *T*. *brucei*, but to different degrees and at different locations. In *L*. *major*, J regulates termination at the end of each PTU to prevent read-through transcription and the generation of RNAs antisense to the genes on the opposing PTU. In contrast, although termination occurs at the end of each PTU in *T*. *brucei* in a J-independent manner, J-dependent termination within a PTU allows developmentally regulated expression of downstream genes.

The core histones H2A, H2B, H3 and H4, package DNA into nucleosomes and represent a critical component of higher order chromatin. All core histones have variant counterparts. Although histone post-translational modifications (PTMs) and their impact on transcription have been well documented, less is known about the role of histone variants in the regulation of transcription [38]. The most understood are variants of H2A and H3. Several variants of H2A exist, including H2A.Z, H2A.B, and macroH2A. Both H2A.Z and H2A.B are associated with transcriptional activation [39–41]. Knockdown of H2A.Z inhibits transcriptional activation [42–44]. Consistent with this, and perhaps the most direct evidence of a transcriptional role of a histone variant, H2A.Z positively correlates with rates of RNAP II elongation, such that the reduction of H2A.Z increases RNAP II stalling [40]. Presumably, the nucleosome destabilizing effect of H2A.Z [45] leads to more accessible DNA at promoter regions for transcription factor binding, as well as promoting RNAP II elongation through gene bodies. Like H2A.Z, H2A.B is enriched at promoter regions and its reduction largely results in the downregulation of gene expression [39, 46]. In contrast, macroH2A is enriched at transcriptionally repressed regions [47] and its reduction results in increased gene expression in an unknown mechanism [48]. Several H3 variants have also been characterized, including H3.3 and CENP-A, both of which are found in most eukaryotes including plants, mammals, and yeast. H3.3 differs from canonical H3 by only 4–5 amino acids and is found predominately at actively transcribed genes, forming more accessible nucleosomes [49–51]. H3.3 also provides a genome stabilization function at repetitive regions such as telomeres and centromeres [49, 52–55]. Recent studies have implicated H3.3 in the maintenance of a repressed chromatin structure [56–58]. Evidence in mouse embryonic stem cells indicates H3.3 is enriched at lowly transcribed developmentally regulated genes where it promotes polycomb repressive complex 2 activity, which catalyzes the formation of the repressive modification H3K27me3 [57, 58]. These findings suggest H3.3 maintains the promoters of developmentally regulated genes in a repressed, but transcriptionally “poised” state important for proper differentiation. H3.3 has also been implicated in the maintenance of H3K9me3 at endogenous retroviral elements in mouse embryonic stem cells [56]. Presence of H3.3 (and H3K9me3) at endogenous retroviral elements repressed retrotransposition and expression of adjacent genes [56]. The centromeric specific histone variant has a well-characterized role in kinetochore formation, but its role in the regulation of transcription, if any, remains unknown [59]. Overall, although much progress has been achieved in the characterization of histone variants, few studies have revealed a direct link between histone variant function and transcriptional regulation.

The J-independent nature of termination at cSSRs in *T*. *brucei* led us to characterize the role of H3.V in regulating RNAP II termination. H3.V and base J co-localize at RNAP II termination sites in *T*. *brucei*, including cSSRs and PTU internal termination sites [13, 14]. H3.V and base J also co-localize at telomeric repeats involved in regulating RNAP I transcription of the *VSG* expression sites [14, 60]. *T*. *brucei* H3.V shares 45% sequence identity with canonical H3, much of the sequence divergence lying within the N-terminus, outside of the histone fold domain. H3.V appears to be unique to kinetoplastids [14, 60] and aside from its localization to termination sites and telomeres, very little is known about H3.V and its potential role in the regulation of transcription termination. We demonstrate here that, similar to phenotypes associated with the loss of J, loss of H3.V leads to defects in RNAP II termination within gene clusters and increased expression of downstream genes. Interestingly, many of the gene expression changes in the *H3*.*V* knockout (KO) are further increased upon the subsequent loss of base J, suggesting that J and H3.V have independent but overlapping roles in regulating transcription termination in *T*. *brucei*. Although the loss of H3.V from cSSRs did not indicate any termination defects leading to transcription of the opposing strand of the adjacent convergent gene cluster, it does lead to increased generation of small interfering RNAs (siRNAs) that map to regions of overlapping transcription. Analysis of nascent RNA suggests this is due to increased transcription of the dual strand siRNA loci at cSSRs. We also detect increased expression of *VSGs* from silent *VSG* ESs in the *H3*.*V* KO, indicating H3.V can act independently in regulating telomeric repression and antigenic variation. Overall these findings provide the first known example of a histone H3 variant that functions as a repressive chromatin mark to promote transcription termination, in this case repressing both mRNAs and non-coding RNAs.

## Results

### H3.V regulates RNAP II transcription at cSSRs

**Fig 1:**
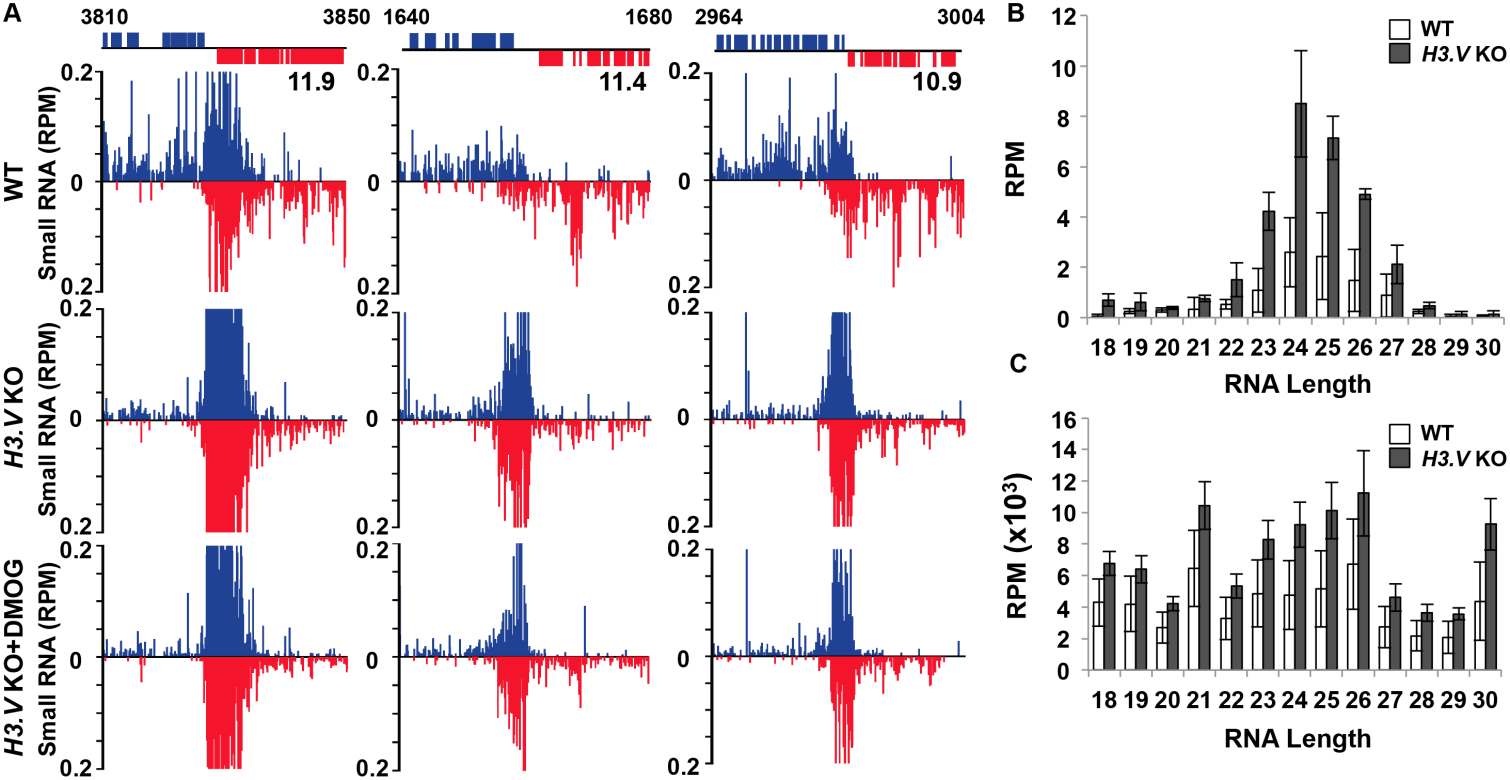
Loss of H3.V stimulates the production of siRNAs in *T*. *brucei*. (A) Small RNA-sequencing reads for three representative cSSRs are shown (11.9, 11.4, and 10.9; where cSSR 11.9 refers to the ninth termination site on chromosome 11) where H3.V loss does not lead to read-through transcription, but does lead to increased siRNAs. Small RNA reads are plotted as reads per million reads mapped (RPM). ORFs and the genomic location (kb) are shown above the graphs. WT: wild type; KO: *H3*.*V* KO; KO+DMOG: *H3*.*V* KO + DMOG. Blue: top strand; red: bottom strand. (B and C) Length distribution of small RNAs. (B) Length distribution of small RNAs from cSSR 11.9. Shown is the RPM for each RNA length observed in cSSR 11.9, a site with a statistically significant increase in 21-27nt RNAs in the *H3*.*V* KO (S2 Table). The average of three independent small RNA-seq experiments is plotted. White bars, WT; black bars, *H3*.*V* KO. Error bars represent the standard deviation. (C) Length distribution of small RNAs genome-wide. The RPM for each RNA length observed in the entire small RNA-seq data set is shown. Data are plotted as in B.

The co-localization of base J and H3.V at RNAP II termination sites in *T*. *brucei* prompted us to examine the role of H3.V in transcription termination. High-throughput sequencing of small RNAs has been shown previously to reveal transcription termination sites in trypanosomatids, as reflected in RNA degradation products [25, 35]. The reduction of base J in *L*. *major* by treatment with DMOG resulted in the production of antisense small RNAs corresponding to genes in the opposing PTU due to read-through transcription at cSSRs [35]. In contrast, we found no evidence of termination defects in *T*. *brucei* at cSSRs following DMOG treatment and the complete loss of J. Antisense small RNAs, indicative of read-through transcription at cSSRs into the downstream PTU, were not increased following the loss of J [35]. We now show that the loss of H3.V also does not result in read-through transcription at cSSRs. No significant changes in antisense small RNAs corresponding to read-through transcription at cSSRs into the downstream PTU were detected by small RNA-seq in the *H3*.*V* KO compared to wild type (WT) *T*. *brucei* (Fig 1A) (small RNA sequencing data discussed in this publication have been deposited in NCBI’s Gene Expression Omnibus and are accessible through GEO Series accession number GSE70229). Subsequent loss of base J in the *H3*.*V* KO parasites, via DMOG treatment, also failed to uncover any defect. These results suggest H3.V is not required to prevent RNAP II read-through transcription at the end of convergent gene arrays in *T*. *brucei*.

Unique to *T*. *brucei*, large peaks of sense and antisense small RNAs map to regions of overlapping transcription at the ends of convergent PTUs at cSSRs ([35]; Fig 1A). These presumably represent the previously characterized Dicer 2-dependent Argonaute-associated siRNAs derived from cSSRs [61]. Consistent with this, we show that the RNAs that map to these regions correspond to the previously characterized siRNA size range in bloodstream form *T*. *brucei* (21-27nt) [61–63] and exhibit characteristic phasing of siRNAs biogenesis, as indicated by the mapping of siRNA sequences in phased intervals at the target loci [64–66] (Fig 1B and S1 Fig). This pattern suggests that these siRNAs were enzymatically processed, most likely by the DCR complex [67], as opposed to random RNA degradation. siRNAs that map to cSSRs are 21-27nt compared to the 18-30nt range of small RNAs genome-wide (Fig 1B and 1C). Although the loss of base J in WT *T*. *brucei* parasites has no effect on siRNA levels [35], the level of siRNAs derived from the cSSRs significantly increased in the *H3*.*V* KO (Fig 1A). To confirm these changes, we repeated the small RNA-seq analysis of WT and *H3*.*V* KO in triplicate. Quantitation of the small RNAs associated with cSSRs genome-wide indicates a statistically significant increase in 21-27nt RNAs at 30 out of 72 cSSRs in the *H3*.*V* KO (Fig 1B and S2 Table). This can be visualized by specifically mapping the siRNA size range of small RNAs (S1A Fig). The increase in siRNAs is not restricted to cSSRs, but occurs at all previously characterized siRNA generating regions of the *T*. *brucei* genome [61, 62]; including the SLACS and ingi retrotransposable elements, CIR147 centromeric repeats, and inverted repeats (S2 Fig). These regions are also enriched with H3.V [14, 68].

**Fig 2:**
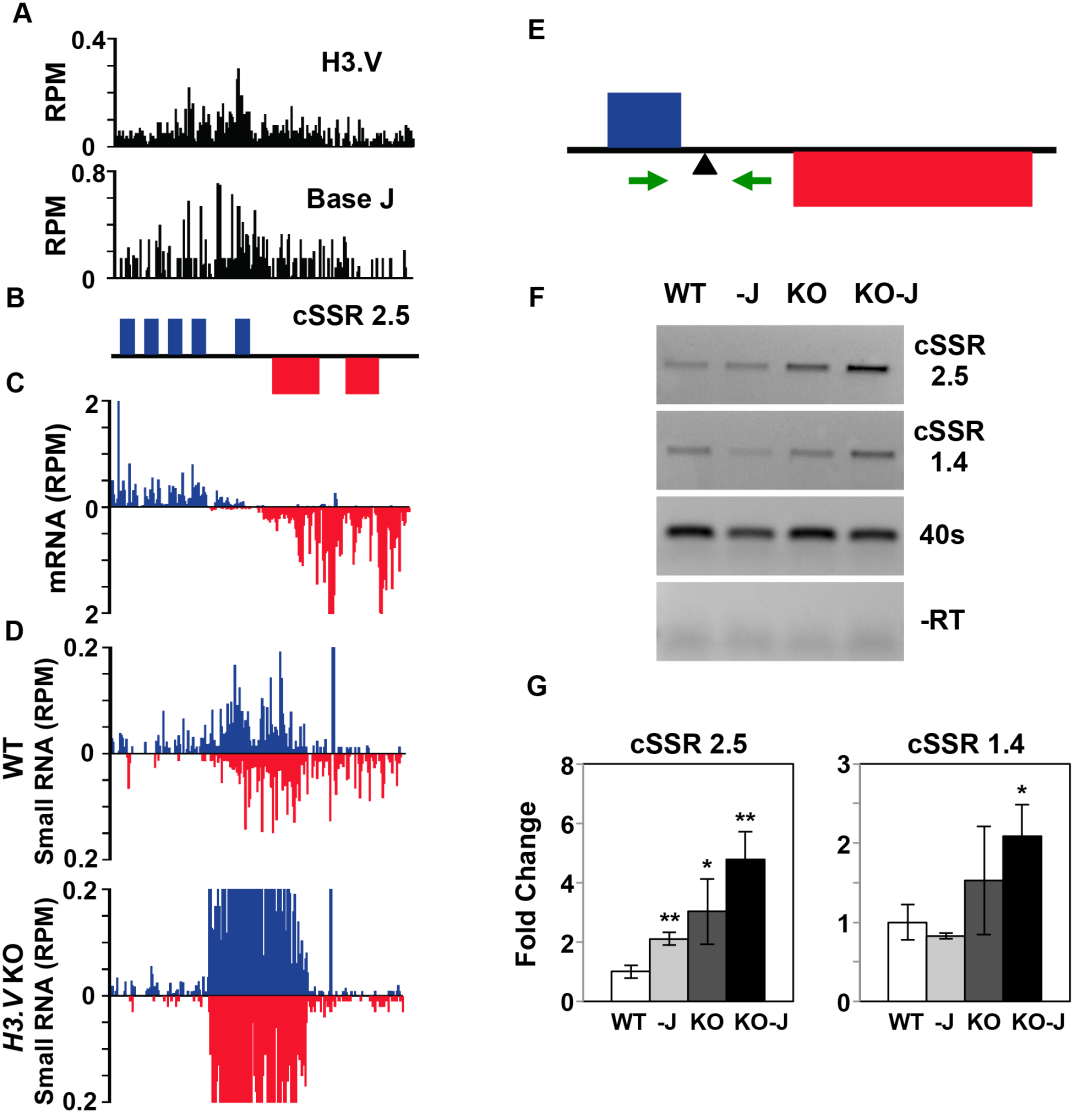
Increased production of nascent RNA in cSSR following the loss of H3.V. A region on chromosome 2 (950–975 kb) representing cSSR 2.5 is shown where H3.V regulates transcription. (A) Base J and H3.V co-localize at sites of RNAP II termination within cSSRs [13, 14]. H3.V ChIP-seq reads and base J IP-seq reads are plotted as reads per million reads (RPM), as previously described [14, 35]. (B) ORFs are shown with the top strand in blue and the bottom strand in red. (C) mRNA-seq reads from wild type *T*. *brucei* are plotted as RPM. Reads that mapped to the top strand are shown in blue and reads that mapped to the bottom strand in red. (D) Small RNA-seq reads from WT and *H3*.*V KO* are mapped as described in Fig 1A. (E-G) Strand-specific RT-PCR analysis of nascent RNA in cSSRs. (E) Schematic representation (not to scale) of primer location and direction at cSSR 2.5 (primers shown as green arrows in this and all subsequent figures). The arrowhead below the line indicates the poly(A) processing site for the final gene in the PTU. (F) Strand-specific RT-PCR analysis. cDNA was synthesized using the reverse primer. PCR was performed using the same reverse primer to make the cDNA plus the forward primer, as indicated. Data is also presented for an additional cSSR on chromosome 1 (cSSR 1.4, 635–637 kb). Wild type: WT; Wild type+DMOG: -J; *H3*.*V* KO: KO; *H3*.*V* KO+DMOG: KO-J. 40S ribosomal protein S11 provides a positive control and minus RT (-RT) negative control is shown. (G) Nested qPCR. Primers were designed within the PCR reaction in F to use in subsequent qPCR analysis. White bars: Wild type; grey bars: Wild type+DMOG; dark grey bars: *H3*.*V* KO; black bars: *H3*.*V* KO+DMOG. All products were normalized to 40S ribosomal protein S11. The average of three independent strand-specific RT-PCR nested qPCR experiments is plotted. Error bars represent the standard deviation. P values were calculated using Student’s t test. *, p value ≤ 0.05; **, p value ≤ 0.01.

Small RNA-seq suggests that the siRNA mapping to dual strand transcription regions at cSSRs is due to continued RNAP II transcription of the convergent PTU resulting in overlapping transcription. An example that illustrates this is shown in Fig 2A–2D, where H3.V (and J) is found enriched at cSSR 2.5. Presumably, H3.V interferes with RNAP II elongation in these dual strand transcription regions. Removal of H3.V would then lead to increased nascent RNA corresponding to these regions (siRNA precursor) that is then processed, resulting in increased siRNA levels that map to both strands (Figs 1 and 2D, S1A and S2 Table). We have previously utilized strand-specific RT-PCR to follow read-through transcription and increased nascent RNA production in *L*. *major* and *T*. *brucei* [35]. Here, primers were designed that span the poly(A) site of the final gene in the PTU, allowing us to specifically detect nascent, unprocessed RNA within cSSR 2.5 (Fig 2E). Strand-specific RT-PCR results indicate an increase in nascent RNA in the *H3*.*V* KO at dual strand transcribed loci at cSSR 2.5 as well as cSSR 1.4 (Fig 2F and 2G). Although no significant increase in siRNAs was observed in DMOG treated WT *T*. *brucei* [35], we do detect a slight increase in nascent RNA following the loss of base J at cSSR 2.5 (Fig 2F and 2G). Nascent RNA is further increased following the loss of both H3.V and J at the two cSSRs analyzed. Overall these results indicate that H3.V, and to a lesser extent J, attenuate transcription elongation within dual strand transcribed loci at cSSRs, potentially enabling regulated expression of siRNAs derived from these sites. Loss of H3.V does not lead to read-through transcription downstream of the dual strand transcribed loci however.

### H3.V inhibits RNAP II elongation within PTUs

**Fig 3:**
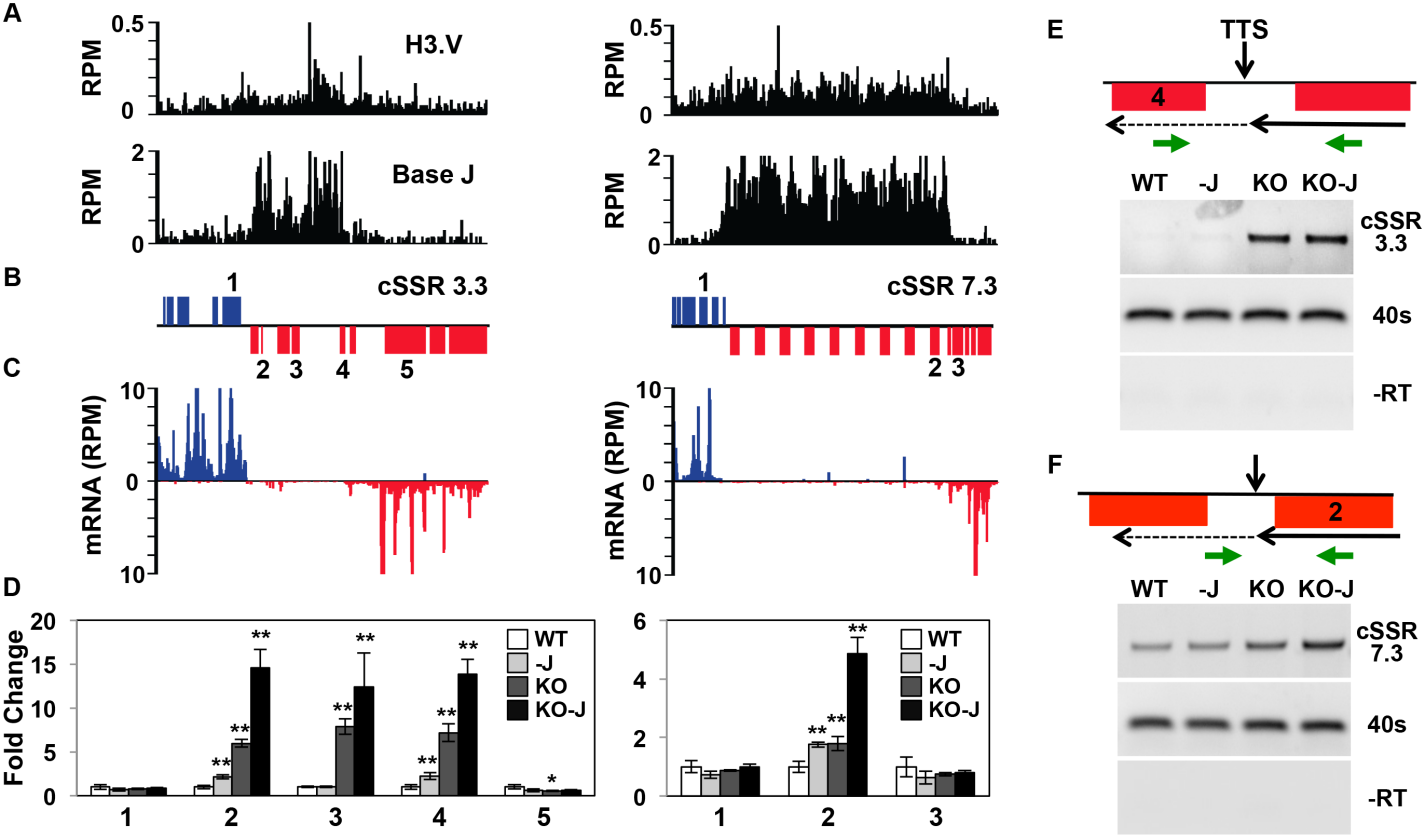
Decreased efficiency of RNAP II termination and increased gene expression following the loss of histone H3.V. A region on chromosome 3 (617–670 kb) representing cSSR 3.3 and chromosome 7 (453–525 kb) representing cSSR 7.3 is shown where H3.V regulates transcription of a cluster of genes. (A-C) Base J and H3.V co-localize at sites of RNAP II termination within a PTU. H3.V ChIP-seq reads and base J IP-seq reads, ORFs, and mRNA-seq reads from wild type *T*. *brucei* are plotted for cSSR 3.3 (left) and cSSR 7.3 (right) as described in Fig 2. (D) RT-qPCR analysis of genes numbered according to the ORF maps above in panel B. As described in Fig 2G, white bars: Wild type; grey bars: Wild type+DMOG; dark grey bars: *H3*.*V* KO; black bars: *H3*.*V* KO+DMOG. Transcripts were normalized against 40S ribosomal protein S11, and are plotted as the average and standard deviation of three replicates. P values were calculated using Student’s t test. *, p value ≤ 0.05; **, p value ≤ 0.01. The silent gene cluster at cSSR 7.3 consists of nine highly similar retrotransposon hot spot protein genes, therefore the primers used to analyze gene 2 also amplify the additional upstream genes. (E and F) Strand-specific RT-PCR analysis of read-through transcription of the two cSSRs analyzed in A-D. Above each panel is a schematic representation (not to scale) of primer location and direction at a transcription termination site (TTS). The vertical arrow indicates the proposed TTS as described in the text [35]. The long solid arrow indicates the direction of transcription and the dashed arrow indicates read-through transcription past the TTS. cDNA was synthesized using the reverse primer (relative to transcription). PCR was performed using the same reverse primer to make the cDNA plus the forward primer, as indicated. 40S ribosomal protein S11 provides a positive control and a minus RT (-RT) negative control is shown.

We have recently shown that base J is present along with H3.V at termination sites within a PTU where J loss results in read-through transcription and increased expression of downstream genes in *T*. *brucei* [35]. Because RNAP II elongation and gene expression is inhibited prior to the end of these PTUs, we refer to this as PTU internal termination. To explore the impact of H3.V on RNAP II elongation at these PTU internal termination sites we analyzed the downstream genes by RT-qPCR in the *H3*.*V* KO cell line. At three representative PTU internal termination sites we detect increased expression of downstream genes in the *H3*.*V* KO (Fig 3A–3D and S3A–S3D Fig). In each of these cases gene derepression is enhanced upon the subsequent loss of J in the *H3*.*V* KO following DMOG treatment (Fig 3D and S3D Fig). Importantly, derepression is limited to genes downstream or within the peak of H3.V/base J. Strand specific RT-PCR using oligos flanking the termination site (based on mRNA-seq and base J localization [13, 35]) detects increased RNA in the *H3*.*V* KO (Fig 3E–3F and S3F Fig). This is consistent with an increase in nascent read-through RNA resulting from continued transcription elongation at PTU internal termination sites marked by base J and H3.V. Consistent with the gene expression changes, read-through is visibly enhanced at region 7.3 upon the subsequent loss of J in the *H3*.*V* KO following DMOG treatment (Fig 3F). These results, combined with our previous study of J regulation of termination [35], suggest J and H3.V have independent and overlapping roles in regulating RNAP II termination through the inhibition of transcription elongation and the expression of downstream genes in *T*. *brucei*.

**Fig 4:**
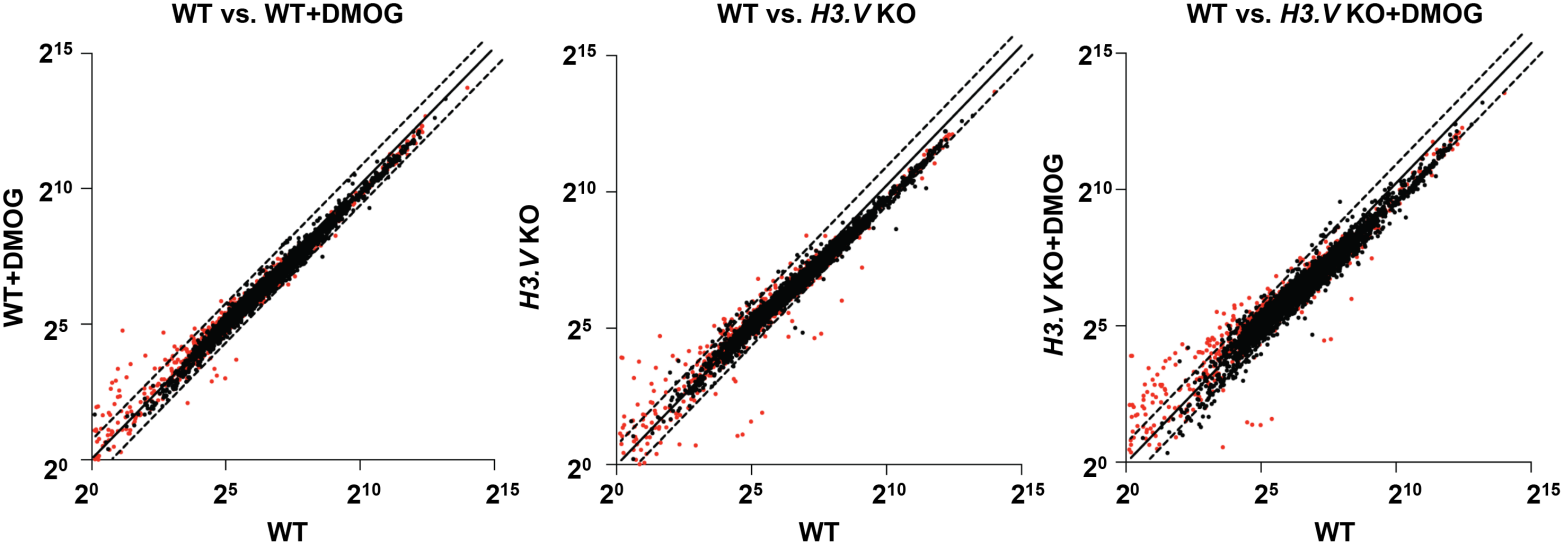
Gene expression changes in the *H3*.*V* KO. The average reads per kilobase per million reads mapped (RPKM) of triplicate mRNA-seq libraries is plotted on a log_2_ scale. Genes differentially expressed by 2-fold or more in *T*. *brucei* following the loss of base J and/or H3.V fall above or below the dotted lines. Red dots indicate genes that are adjacent to H3.V (S1 Table). Only mRNAs with an RPKM≥1 are included.

**Fig 5:**
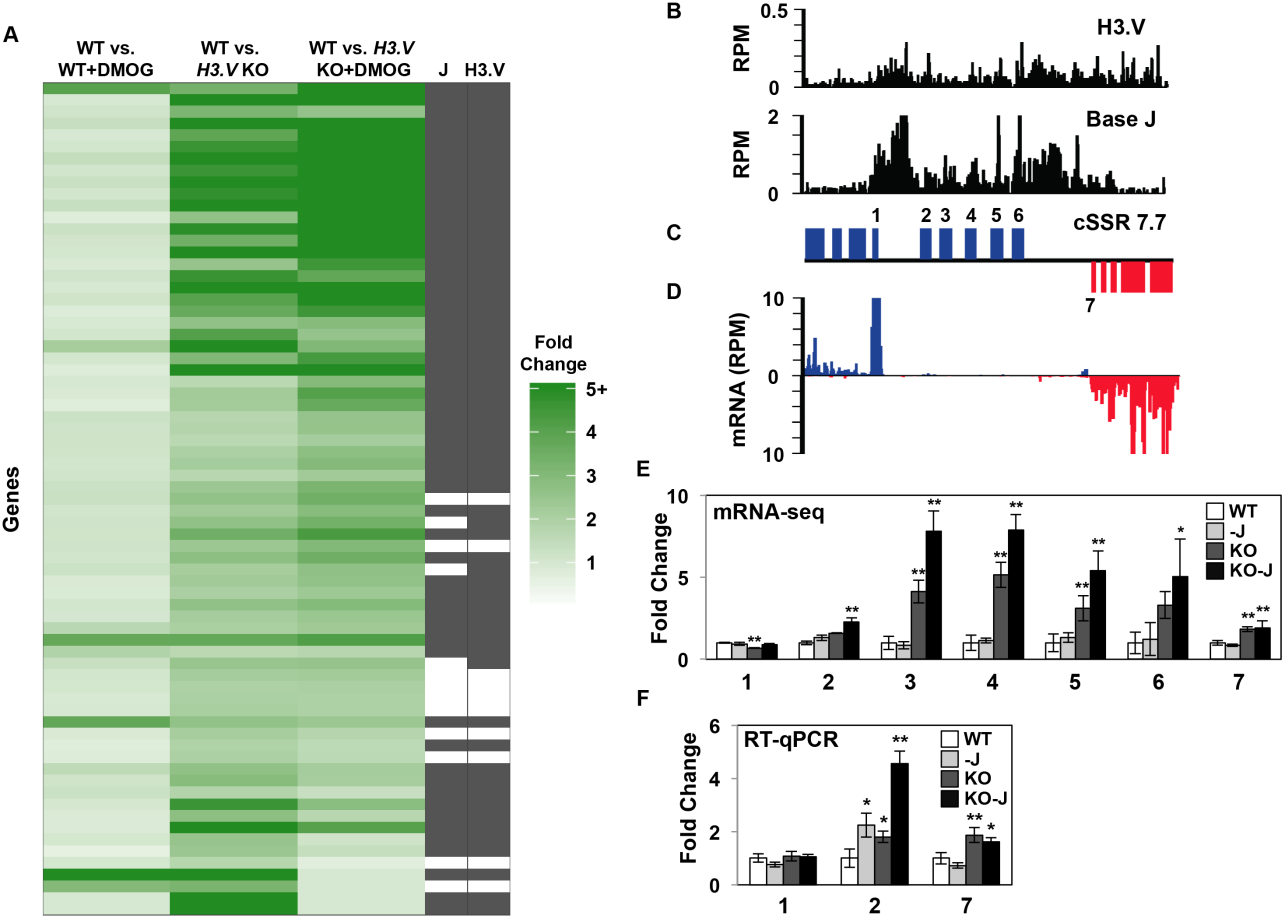
H3.V and base J have independent yet additive roles in regulating termination and gene expression. (A) Heatmap of genes upregulated in the *H3*.*V* KO. For the list of genes represented on the heatmap see S1 Table. Clustering of genes at the top indicate those that are further upregulated upon loss of base J in the *H3*.*V* KO. J and H3.V columns indicate whether each gene is located within 10 kb of the modification (filled black box), as described in the Materials and Methods section. (B-D) H3.V/J localization, gene map, and mRNA-seq reads plotted for a gene cluster on chromosome 7 at cSSR 7.7 (position 1750–1800 kb shown) is illustrated as described in Fig 3. (E) Plot of the mRNA-seq data for the genes indicated (numbered) in the ORF map. The average RPKM of triplicate mRNA-seq libraries was used to determine fold changes, with wild type set to 1. Error bars indicate the standard deviation between mRNA-seq replicates and p values, determined in Cuffdiff, are indicated by asterisks: *, p value ≤ 0.05; **, p value ≤ 0.01. (F) RT-qPCR analysis of gene expression for the indicated genes (according to the ORF map) as described in Fig 3D. White bars: Wild type; grey bars: Wild type+DMOG; dark grey bars: *H3*.*V* KO; black bars: *H3*.*V* KO+DMOG. P values were calculated using Student’s t test. *, p value ≤ 0.05; **, p value ≤ 0.01.

To further explore the role of H3.V in the regulation of termination and whether H3.V functions similarly across the genome, we performed mRNA-seq to compare the expression profiles of WT, WT+DMOG, *H3*.*V* KO and *H3*.*V* KO+DMOG cells (GEO accession number GSE69929). This led to the detection of 153 mRNAs that are increased at least 2-fold in one or more of the treatments (S1 Table). Many of the gene expression changes have been confirmed by RT-qPCR (S4 Fig and below). Consistent with our previous mRNA-seq results, in WT cells treated with DMOG we observe similar increases in the expression of genes downstream of base J (and H3.V), which we previously demonstrated is caused by an RNAP II transcription termination defect within a PTU [35]. However, we now see that a significant number of genes downstream of J/H3.V within other PTUs are upregulated following the loss of H3.V, and that many are further increased following the subsequent loss of J (Fig 4, S1 and S4 Tables). In the *H3*.*V* KO we identified 71 genes that are upregulated (Fig 5A and S1 Table). Although many of these genes are not increased by 2-fold in the WT+DMOG condition, some respond at least slightly to the loss of J in WT cells: 28 of the 71 genes upregulated in the *H3*.*V* KO are also increased at least 1.3-fold in the WT+DMOG condition, suggesting J and H3.V have overlapping functions in regulating termination at these sites, where H3.V plays a dominant role. Consistent with this, 42 of the 71 H3.V regulated genes are upregulated even further upon subsequent loss of base J in the *H3*.*V* KO. This trend is evident in the heatmap shown in Fig 5A. A specific example is shown in Fig 5B–5F where a cluster of genes is affected by the loss of H3.V, and to a lesser extent base J. Both chromatin marks are enriched upstream of and within this gene cluster, which consists of genes annotated as *VSG* pseudogenes, an atypical *VSG*, and a hypothetical protein. mRNA-seq indicates gene upregulation is largest in the absence of H3.V and J, which we confirmed by RT-qPCR (Fig 5E and 5F). An additional example is shown in S5 Fig Chromosome maps indicating the genomic location of upregulated genes upon the loss of H3.V and J are shown in S6 Fig. These results also confirm our initial analyses of nascent and steady-state RNA indicating a role for H3.V (and J) regulating termination and expression of downstream genes (Fig 3 and S3 Fig).

80% of the affected genes are found within 10 kb of H3.V and base J (see S1 Table and S7 Fig) and thus fit a model where derepression occurs as a result of deregulated transcription elongation/termination within a PTU following the loss of the two chromatin marks. In support of this model, strand-specific RT-PCR analysis links gene derepression with increased nascent RNA production downstream of J/H3.V marks within the PTU (Figs 3E–3F and S3F). In a few cases, genes that are further than 10 kb downstream of base J and H3.V are still within a regulated cluster, explaining why some genes are indicated as not adjacent to J/H3.V but can still be regulated by these epigenetic marks (e.g. genes Tb427.07.6730-Tb427.07.6780, S1 and S4 Tables). Although clusters of genes downstream of a J and H3.V enriched region are similarly upregulated in many cases, the 153 upregulated genes localize to 91 different PTUs (S4 Table). Therefore, H3.V and J repress gene expression genome-wide, often repressing the expression of a single gene, usually the last gene, within a PTU. Overall, the data indicate that base J and H3.V have independent but overlapping roles in regulating RNAP II transcription termination within specific PTUs and enable regulated expression of downstream genes.

Although the majority of the differentially expressed genes are upregulated, we also see some downregulated (S1 Table). 35 genes are downregulated by at least 2-fold in the *H3*.*V* KO cells. Unlike the upregulated genes, where many were increased furthest following the combined loss of J and H3.V, only 7 of the 35 downregulated genes decreased more in the *H3*.*V* KO+DMOG condition. However, 33 of the 35 downregulated genes are found proximal (within 10kb) to H3.V and J. The effect is also locus specific and mainly restricted to arrays of genes transcribed from the same strand. These points indicate a strong link between H3.V/J localization and gene expression changes observed. A few examples are indicated in S8 Fig. Among the genes with most significant downregulation upon loss of H3.V were procyclins and procyclin associated genes (PAGs), which include surface protein encoding genes most highly expressed during the (procyclic) insect stage of the parasite [69]. Although PAGs are located in multiple copies in the genome, within RNAP II transcribed arrays or within RNAP I transcribed procyclin arrays [69, 70], the PAGs downregulated following the loss of H3.V (PAG1, PAG2, PAG4 and PAG5) are specifically arranged in an RNAP I transcribed array. For example, there are two PAG2 genes located on chromosome 10, one in an RNAP II transcribed PTU and the other in the RNAP I transcribed procyclin locus. The only gene that is significantly downregulated when H3.V is deleted is the one within the RNAP I procyclin locus. A similar locus specific alteration of PAG expression was seen upon the depletion of histone H1 [71]. Interestingly, the PAGs within this locus undergo overlapping RNAP II and RNAP I transcription, i.e. continued RNAP II transcription of the upstream opposing PTU produces antisense PAG RNAs [72]. This suggests a possible mechanism of PAG (and procyclin) downregulation resulting from increased formation of dsRNAs upon the loss of H3.V (see discussion). Similarly, other downregulated genes are arranged in opposing transcriptional genes pairs where extended RNAP II transcription would result in dsRNA for each mRNA (S8 Fig). We also identified 25 genes that are downregulated specifically in the *H3*.*V* KO+DMOG condition, though 22 of these genes are not located near H3.V or J. We therefore assume many of these changes are an indirect effect of genes that are upregulated in this cell line. For example, we have demonstrated that the genome-wide increase in RNAP II transcription in *T*. *cruzi* results in a global increase in gene expression that includes proteins that degrade specific mRNAs [24].

### H3.V regulates expression of RNAP I transcribed *VSG* genes

**Fig 6:**
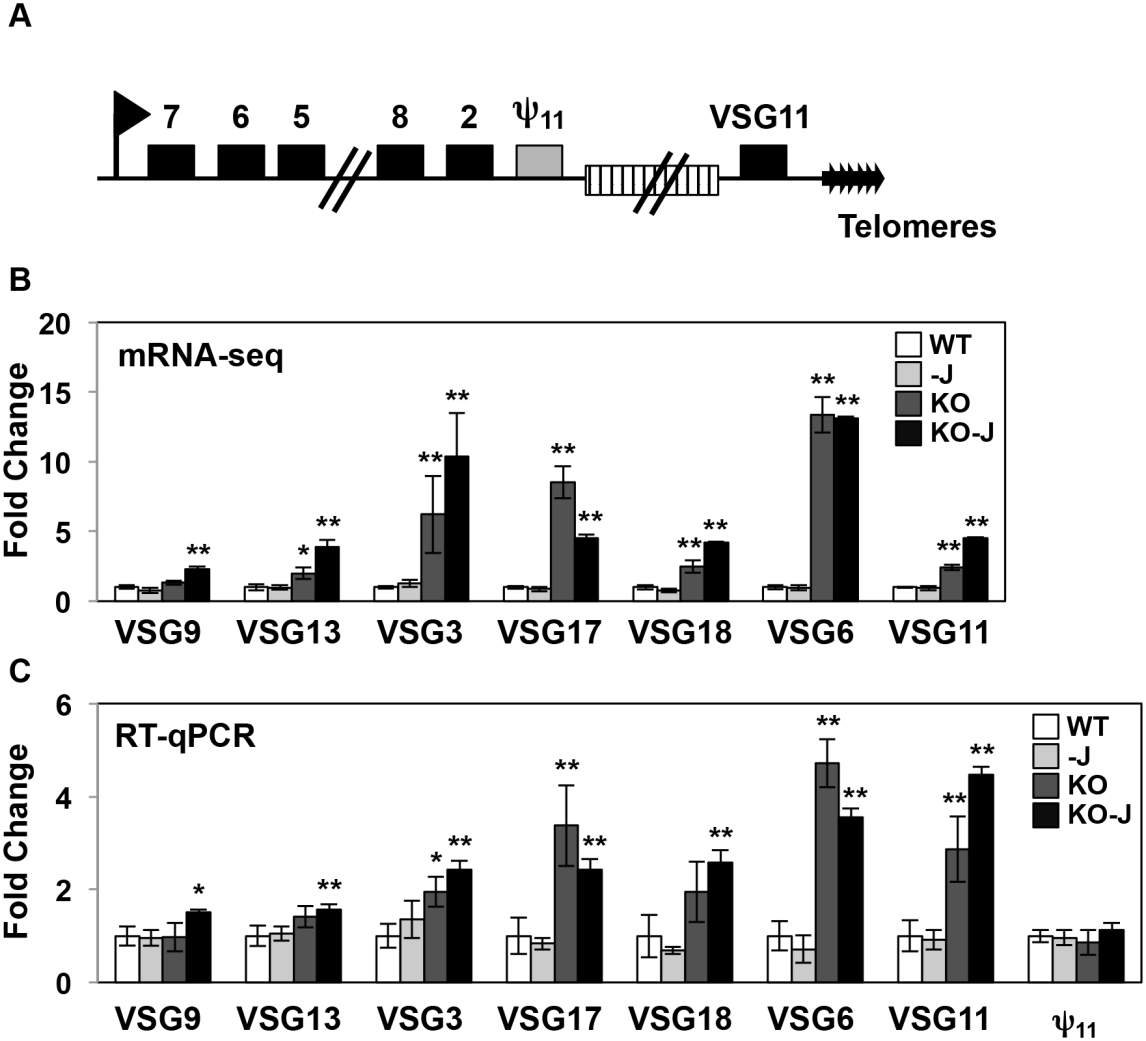
H3.V regulates *VSG* gene expression from silent telomeric bloodstream expression sites. (A) A schematic diagram of the silent ES15 (not to scale). The box with stripes represents the 70 bp repeats. Numbers indicate *ESAG* genes. Grey box represents the *VSG* pseudogene 11 (Tb427.BES126.13). (B-C) mRNA-seq and RT-qPCR analysis of the indicated *VSG* genes in silent expression sites. As described in Figs 3D and 5E, white bars: Wild type; grey bars: Wild type+DMOG; dark grey bars: *H3*.*V* KO; black bars: *H3*.*V* KO+DMOG. For mRNA-seq analysis, p values determined by Cuffdiff are indicated by asterisks: *, p value ≤ 0.05; **, p value ≤ 0.01. For RT-qPCR analysis, p values were calculated using Student’s t test. *, p value ≤ 0.05; **, p value ≤ 0.01.

In addition to RNAP II termination sites, H3.V co-localizes with base J at telomeres, which in the *T*. *brucei* genome contain the RNAP I transcribed polycistronic units involved in antigenic variation (so called *VSG* expression sites, ESs) (Fig 6A) [27, 28]. mRNA-seq results indicate that the deletion of H3.V leads to increased expression of *VSG* genes from silent ESs, which we have confirmed by RT-qPCR (S1 Table and Fig 6B). Although *VSG*s within expression sites are not affected by the loss of J in WT cells, five of the *VSG*s are further upregulated upon the loss of J in the *H3*.*V* KO (S1 Table and Fig 6B).

mRNA-seq indicates several expression site associated genes (*ESAGs*) within silent ESs with differential expression. Because the repetitive nature of *ESAGs* complicates read alignment of mRNA-seq data, we analyzed several genes by RT-qPCR using primers that are specific to ES *ESAGs* (S9 Fig). Consistent with mRNA-seq results, with the apparent exception of *ESAG8*, most of the *ESAGs* are not upregulated in the *H3*.*V* KO (S9 Fig). The lack of significant change in the majority of *ESAGs* within the ESs suggests derepression of telomere-proximal *VSG* genes after H3.V depletion is not due to transcriptional activation of silent promoters. Repression of silent ESs is mediated in part by the inhibition of RNAP I elongation within the ES preventing the production of *VSG* mRNA from the silent ESs [73]. Similar to its inhibition of RNAP II transcription elongation at termination sites within PTUs genome-wide, H3.V may function at telomeric regions to attenuate transcription elongation within the silent ESs, thereby preventing transcription of silent *VSGs*. To further investigate this possibility and determine how telomere-proximal and telomere distal genes are affected after loss of H3.V, we used RT-qPCR to compare the derepression of a unique gene in the silent ES15 that is located 10 kb (*VSG* pseudogene 11, Tb427.BES126.13) and 1 kb (*VSG11*) upstream of the telomere (Fig 6; [74]). Although *VSG11* was upregulated in the *H3*.*V* KO, we found that the *VSG* pseudogene is not significantly derepressed in the *H3*.*V* KO or upon the loss of H3.V and J (Fig 6B). These results overall suggest H3.V is involved in the repression of *VSGs* in silent ESs, but does not significantly impact silencing of the entire ES, similar to the role of telomere localized TbRAP1 [75]. *ESAG8* derepression is consistent with ES derepression from the promoter, however upregulation of multiple *ESAGs* would be expected if derepression of the silent ESs occurs from the promoter, which was observed after ISWI depletion [76]. Although we cannot rule out possible alterations in VSG switching in the *H3*.*V* KO (see discussion), the data here suggest H3.V is involved in repressing RNAP I transcription of *VSGs* within the silent ESs as well as RNAP II transcription of *VSG* genes within genome internal PTUs.

## Discussion

H3.V is a kinetoplastid-specific H3 variant and appears to be the only H3 variant found in these early-diverged eukaryotes. The *T*. *brucei* H3.V shares only 45% sequence identity with the canonical H3 [60]. Although H3.V localizes to centromeres, it is not essential for viability and does not contain sequence variations common to all identified centromeric H3 variants [14, 60, 68]. Aside from its localization to RNAP II termination sites and telomeres, the functional significance of H3.V and its potential role in the regulation of RNAP II termination has been unexplored. We have demonstrated that H3.V negatively regulates transcription elongation and promotes RNAP II termination in *T*. *brucei*. Several lines of evidence support this conclusion. At many of the same sites within gene clusters where we have previously shown J regulates transcription termination and expression of downstream genes, we detect similar increases in the expression of genes downstream of the termination site following the loss of H3.V. Also similar to J loss, detection of increased nascent, unprocessed RNA by strand-specific RT-PCR at these sites supports the conclusion that the loss of H3.V leads to read-through transcription. Furthermore, the loss of H3.V from regions where dual strand transcription naturally occurs at cSSRs leads to increased levels of siRNAs, and strand-specific RT-PCR indicates this is due to increased transcription of the cSSR. These findings overall suggest H3.V imparts a repressive chromatin structure that is refractory to transcription elongation, or potentially recruits other repressive factors or transcription termination factors at RNAP II termination sites. To our knowledge, this is the first example of a histone H3 variant that has been shown to repress the expression of mRNAs and non-coding RNAs by promoting transcription termination.

These results extend our previous findings that base J functions to prevent read-through transcription at termination sites within gene clusters in *T*. *brucei*, revealing an overlapping role of J and H3.V in the regulation of transcription termination. However, H3.V appears to have a broader and in many cases a more dominant role. In this study mRNA-seq indicated 71 genes were upregulated by 2-fold or more following the loss of H3.V. 39% of those genes were also affected by at least 1.3-fold following the loss of J alone, and 59% were further increased upon the subsequent loss of base J in the *H3*.*V* KO. Thus H3.V and J appear to function similarly, but independently in the regulation of transcription termination. Although we cannot exclude a potential role of H3.V (and J) in the regulation of RNA processing, the increase in both unprocessed (nascent) and processed RNAs (mRNAs and siRNAs) strongly suggests H3.V regulates RNA abundance at the level of transcription and that the defects we observe are not simply due to an alteration of RNA processing. We propose a model in which both H3.V and base J inhibit RNAP II elongation, and therefore stimulate termination at sites within gene clusters (S10 Fig). According to this model, the loss of J within a gene cluster results in read-through transcription and expression of genes that were previously silent, but the presence of H3.V within the downstream cSSR prevents increased dual strand transcription and thus siRNAs are not significantly increased. Similarly, the loss of H3.V leads to read-through transcription at termination sites within a gene cluster and subsequent gene derepression. H3.V loss also results in increased transcription of dual strand transcribed loci at cSSRs, giving rise to more siRNAs. Therefore, base J is a chromatin modification that specifically regulates the expression of a subset of genes in the bloodstream form of *T*. *brucei* parasites, whereas H3.V regulates a similar, but larger subset of genes, in addition to the generation of siRNAs.

Recent description of the trypanosome stress response has indicated that the location of a gene within a PTU can impact its expression, presumably via regulated transcription elongation [77]. Here we provide evidence that regulated transcription and expression of genes within PTUs can be achieved through their spatial organization and position relative to H3.V and base J. However, the biological significance of the gene expression changes we describe following the loss of these chromatin marks, remains unclear. It does not help that the majority of the regulated genes are annotated as hypothetical proteins of unknown function. Many of the H3.V and base J regulated genes include *VSGs*, *ESAGs*, *RHS* proteins, and pseudogenes that are normally lowly expressed (or not at all) in wild type *T*. *brucei*. Interestingly, consistent with base J synthesis, *VSGs* and *ESAGs* are developmentally regulated, typically exclusively expressed in bloodstream form trypanosomes from the telomeric PTU (*VSG* ESs). Monoallelic expression of a *VSG* ES leads to the expression of a single VSG on the surface of the parasite, a key aspect of trypanosome antigenic variation. Therefore the repression of silent *VSGs* by H3.V/J allows the parasite to maintain this monoallelic expression. Another important aspect of antigenic variation is the periodic switching of the VSG protein expressed on the surface allowing the parasite to remain a step ahead of the host immune response. DNA recombination (i.e. gene conversion events) of silent *VSG* genes into the active ES is the dominant driver of trypanosome antigenic variation. Transcription of a donor DNA sequence has been shown to increase its use during gene conversion events in human cells [78]. It has also been demonstrated that active transcription in *T*. *brucei* stimulates DNA recombination [79]. Therefore, the regulation of transcription of silent *VSGs* by H3.V/J, including *VSGs* in silent telomeric ESs and *VSG* pseudogenes at the end of genomic internal PTUs, could play a role in gene conversion events. It is well characterized that during late phases of mammalian infection, trypanosomes predominately express mosaic *VSGs* comprised of multiple *VSGs* and pseudogenes [80]. These findings thus raise the possibility that regulated transcription of silent *VSGs* by H3.V/J, in particular *VSG* pseudogenes at internal PTUs, contributes to gene conversion events that result in the formation of these mosaic *VSGs*.

If H3.V and base J are utilized to effect specific gene expression changes, then mechanisms likely exist to overcome the silencing effects of these modifications, i.e. regulated addition and/or removal. Histone chaperones and chromatin remodeling complexes incorporate histone variants at specific chromatin locations. Thus, regulation of (unidentified) histone chaperones that incorporate H3.V could enable regulated gene expression. Chromatin remodeling proteins could also be involved in the removal of H3.V. The first step of J synthesis consists of thymidine oxidation by JBP1 and 2, which utilize oxygen and 2-oxoglutarate and require Fe2+ as a cofactor. Changes in oxygen concentrations or metabolic changes could thus impact J synthesis and effect gene expression changes. We previously demonstrated oxygen regulation of JBP1/2 and J synthesis, which led to changes in gene expression and pathogenesis of *T*. *cruzi* [24, 31]. JBP1/2 have been shown to have differential chromatin substrates for de novo J synthesis in vivo [13]. Therefore, regulation of JBP1/2, or associated factors, could provide differential regulation of J synthesis at specific loci. Reiterative oxidations of thymidine residues by JBP1/2 [29], similar to TET mediated oxidation of cytosines [81], may also contribute to regulated J synthesis at specific loci.

Loss of H3.V also led to derepression of *VSG* genes within the silent telomeric ESs. We hypothesize, similar to its effect on RNAP II termination within PTUs genome-wide, H3.V localized to telomeric repeats limits basal levels of RNAP I transcription elongation within silent ESs [73]. A similar telomeric VSG derepression effect was observed following the loss of RAP1 in *T*. *brucei* [75]. The lack of significant *ESAG* gene derepression suggests loss of H3.V does not result in derepression from the promoters of silent ESs, though *ESAG8* upregulation is consistent with this possibility. Therefore, we acknowledge that further detailed analysis is required, including the use of tagged silent ESs, to fully understand the role of H3.V on ES transcription. Because of our inability to effectively measure VSG switching rates in our *H3*.*V* KO cell line, we also cannot exclude the possibility that H3.V restricts VSG switching, though a recent study has indicated that switching frequency does not appear to change significantly in the *H3*.*V* KO or upon the loss of H3.V and J (Schulz, Papavasiliou, and Kim, personal communication). While base J has no apparent independent role in telomeric repression or VSG switching, the additional derepression of *VSG* genes observed in the *H3*.*V* KO upon loss of J suggests the novel modified base can act synergistically with H3.V in telomeric silencing and antigenic variation. This function is consistent with the distinct localization of base J in the silent ESs, with J density highest close to the telomeres [82].

We also found a specific RNAP I transcribed procyclin gene cluster that was downregulated following the loss of J and even more so by the loss of H3.V. As mentioned above, this locus has been shown to undergo overlapping RNAP II and RNAP I transcription in the procyclic form *T*. *brucei* [72]. In the procyclic form, transcription from the opposite strand was detectable from GU2 to EP1 (S8 Fig). Increased antisense transcription from the RNAP II PTU on the opposite strand led to antisense RNA and co-transcriptional silencing of PAG genes [72]. In contrast, there was little transcription from the opposing strand in bloodstream forms. The presence of H3.V, and to some extent J, may inhibit this dual strand transcription in bloodstream forms, thus reducing the formation of dsRNA and/or transcriptional interference by RNAP II that could interfere with the expression of the procyclin locus. However, analysis of this locus in procyclic cells has indicated that the loss of Argonaute did not appear to alter the expression of the procyclin genes [83], thus the role of dsRNAs at this locus, if any, remains unknown. Interestingly, several of the other genes that are downregulated in the *H3*.*V* KO are arranged in opposing transcriptional gene pairs where extended RNAP II transcription would result in dsRNA for each mRNA (S8 Fig). Additional studies are needed to characterize the role of H3.V in regulating transcription at these loci. As we described for the effect of base J in *T*. *cruzi* [24], it is also possible that some transcripts are downregulated in the *H3*.*V* KO due to secondary effects of derepressed genes, which could include regulatory proteins (destabilizing specific mRNAs).

Future studies are necessary to further elucidate the mechanisms by which H3.V regulates transcription, including what proteins interact with H3.V, the impact of H3.V on nucleosome structure/stability, whether H3.V undergoes any post-translational modifications, and how chromatin structure is affected by the loss of H3.V. It is not clear how sequence variation in H3.V confers its specific localization to transcription termination sites or its function. H3 PTMs have not been well characterized in *T*. *brucei*, however those identified by mass spectrometry analyses include S1 and K23 acetylation and K4, K32, and K76 methylation [84]. In comparison to the canonical H3, the *T*. *brucei* H3.V differs in that it contains an A1 and R23, and thus lacks the corresponding acetylation. However, differences in PTMs between H3.V and H3 have not been investigated.

Interestingly, the loss of H3.V and base J in *T*. *brucei* did not result in read-through transcription that extends into the downstream gene cluster encoded on the opposite strand and generation of antisense RNAs. In *L*. *major* the loss of J alone led to such read-through transcription at a majority of the cSSRs sites in the genome [35] whereas the loss of H3.V had no effect [85]. Overall these findings indicate that the function of epigenetic modifications in kinetoplastid parasites is not necessarily conserved. One obvious difference between *T*. *brucei* and *L*. *major* is the absence of a complete RNAi pathway in *L*. *major* [86, 87]. It would therefore be interesting to investigate the role of H3.V in regulating siRNAs in a *Leishmania* species with an intact RNAi pathway.

In addition to its role in the regulation of dual strand transcription at cSSRs and the generation of siRNAs, we also find H3.V regulates other characterized siRNA generating loci, including the SLACS and ingi retrotransposable elements, CIR147 centromeric repeats, and inverted repeats. Aside from the SLACS and ingi derived siRNAs, the function of siRNAs in *T*. *brucei* is unclear. Mature SLACS and ingi transcripts are present at low levels in WT *T*. *brucei* due to the presence of a functional RNAi pathway [88]. Surprisingly, despite the increase in SLACS and ingi siRNAs following the loss of H3.V, we also observe a modest increase in SLACS (*Tb427tmp*.*211*.*5010*) and ingi transcripts by mRNA-seq (S1 Table). Although the relative increase in steady-state level of siRNAs is greater than that of the mature mRNA transcript, presumably the increased transcription of these loci in the absence of H3.V increases both RNA species. Dicer 2 is responsible for the formation of siRNAs derived from cSSRs, and its removal (and corresponding decrease in siRNAs) did not have a significant effect on the expression of genes located at cSSRs that coincide with the siRNA peak [61], suggesting that cSSR derived siRNAs do not regulate mRNA abundance. Consistent with this, at cSSRs where we detect increased siRNAs we do not observe significant decreases in mRNAs from genes that overlap the siRNA peak. Therefore the function, if any, of cSSR derived siRNAs remains unknown.

In summary, we have provided evidence for the connection between a histone H3 variant and transcription termination for the first time. These findings highlight the importance of chromatin modifications in the regulation of transcription termination, particularly in early-diverged eukaryotes with unique polycistronic transcription. These findings also have direct implications for a strictly post-transcriptional model of gene expression regulation in kinetoplastids.

## Materials and Methods

### Parasite cell culture

WT and *H3*.*V* KO bloodstream form *T*. *brucei* 221a cell lines of strain 427 were cultured in HMI-9 medium as described previously [89]. The bloodstream form *T*. *brucei H3*.*V* KO cell line, generated by deleting both H3.V alleles by homologous recombination [60], was provided by George Cross. DMOG treatment of cells was performed by supplementing media with 1mM DMOG for 5 days as described previously [35].

### Strand-specific RNA-seq library construction

Small RNA-sequencing was performed using two different methods. The analysis of WT, *H3*.*V* KO, and *H3*.*V* KO+DMOG (Figs 1A, 2D and S2 Fig) was performed as previously described [35]. Briefly, small RNAs were isolated from *T*. *brucei* (5x10^7^ cells) using a Qiagen miRNeasy kit according to the manufacturer’s instructions. The small RNA-seq libraries were prepared using approximately 250ng small RNA by Vertis Biotechnology AG, Germany. The small RNA sample was poly(A)-tailed using poly(A) polymerase. Then, the 5'PPP and cap structures were removed using tobacco acid pyrophosphatase (TAP, Epicentre). Afterwards, an RNA adapter was ligated to the 5'-monophosphate of the RNA. First-strand cDNA synthesis was performed using an oligo(dT)-adapter primer and the M-MLV reverse transcriptase. The resulting cDNAs were PCR-amplified to about 10–20 ng/μL using a high fidelity DNA polymerase. The cDNAs were purified using the Agencourt AMPure XP kit (Beckman Coulter Genomics). Quality and concentration of all libraries was determined by capillary electrophoresis and high throughput sequencing was performed on a HiSeq2000 (Illumina). Sequencing reads were mapped to the *T*. *brucei* reference genome using Bowtie2 version 2.2.3 with local sensitive mode, all other parameters default, [90] and further processed using Samtools 1.2 [91]. Reads shorter than 18 bp were discarded before mapping. Genome and gene annotations of strain 427 version 6.0 were downloaded from EuPathDB [92] and used as the reference in all small RNA-seq analyses. RPM were calculated using a window size of 101 bp and a step size of 101 bp. Total sequence reads and overall alignment rate for all RNA-seq libraries discussed in this publications are listed in S3 Table.

Small RNA-sequencing of the triplicate analysis of WT and *H3*.*V* KO (Figs 1B, 1C, S1A and S2 Tables) was performed in a similar manner. Briefly, total RNA was isolated from log phase *T*. *brucei* cultures (5x10^7^ cells) using Trizol according to the manufacturer’s instructions. The small RNA-seq libraries were prepared using approximately 250ng total RNA using the Illumina-compatible NEBNext small RNA library preparation kit following the manufacturer protocol (New England Biolabs). Quality and concentration of all libraries was determined using a Bioanalyzer 2100 (Agilent). Libraries were pooled using equi-molar amounts and sequenced on a NextSeq500 (Illumina). Both library construction and sequencing were done at the Georgia Genomics Facility (GFF). Small RNA reads were quality and adapter trimmed using Cutadapt [93] and reads shorter than 18 nucleotides were discarded. Reads were mapped to the *T*. *brucei* reference genome using Bowtie2 version 2.2.3 with the following parameters “-a -D 10 -R 5 -N 1 -L 15 -i S,1,0.50”[90] and further processed using Samtools 1.2 [91], BEDTools [94], and custom scripts. RPM shown in Fig 1B and 1C were calculated by dividing the total number of reads in each size class by the total million reads mapped. For Fig 1B, only reads that mapped to the dual strand transcribed region on cSSR 11.9 (3826–3835 kb) were included, whereas Fig 1C includes all mapped reads. Differential expression analysis on small RNA-seq read count data (WT versus *H3*.*V* KO) was performed using EdgeR (S2 Table). Significance testing was pairwise using Fisher’s Exact test. Significance was assessed in both the total small RNA-seq reads and in the 21-27nt reads.

For the mRNA-seq, total RNA was isolated from log phase *T*. *brucei* cultures (5x10^7^ cells) using Trizol. 12 mRNA-seq libraries were constructed (triplicate WT, WT+DMOG, *H3*.*V* KO, and *H3*.*V* KO+DMOG) using Illumina TruSeq Stranded RNA LT Kit following the manufacturer’s instructions with limited modifications. The starting quantity of total RNA was adjusted to 1.3 μg, and all volumes were reduced to a third of the described quantity. High throughput sequencing was performed at the Georgia Genomics Facility (GFF) on a NextSeq500 (Illumina). Raw reads from mRNA-seq were first trimmed using Trimmomatic version 0.32 [95]. The single-end reads were trimmed for TruSeq3 adapters; leading and trailing bases with quality less than 15 and reads with average quality less 20 were removed. Finally, any reads shorter than 50 base pair were discarded. Remaining reads were locally aligned to the *T*. *brucei* Lister 427 version 9.0 genome, from EuPathDB [92], using Bowtie2 version 2.2.3 [90]. All settings were default except specifying sensitive local and further processed with Samtools 1.2 [91]. Transcript abundances were computed using the Cufflinks suite version 2.2.1 [96]. For individual replicates, Cuffnorm was used with the library type fr-firststrand flag and the *T*. *brucei* Lister 427 version 9.0 annotation (downloaded from EuPathDB [92]). To estimate gene expression levels for a condition, replicates were used together and analyzed by Cuffdiff with the *T*. *brucei* Lister 427 version 9.0 annotation. Default parameters were used except specifying library type fr-firststrand. All p values reported here, determined by Cuffdiff, reflect the FDR-adjusted p value. Correlation coefficients for mRNA-seq replicates of WT, WT+DMOG, and *H3*.*V* KO were all greater than 0.96 and *H3*.*V* KO+DMOG replicates were greater than 0.91. To express the transcripts levels for individual mRNA encoding genes as shown in S1 Table, we determined the number of reads per kilobase per million reads (RPKM) [97]. Briefly, we counted the number of reads mapped to all annotated transcriptomic features (e.g. mRNA) on the same strand (i.e. sense) and opposite strand (i.e. antisense). Both the sense and antisense read numbers were normalized by length of the feature (in kilobase) and the total number of reads (in millions) mapped to non-structural RNAs in the corresponding library (i.e. number of mappable reads excluding rRNA and tRNA reads). mRNA-seq data shown in Figs 2C, 3C, 5D, S3C and S5C are from our previously published dataset [35] and are consistent with mRNA-seq performed in this study (see S3 Table for the RNA-seq datasets used in each figure). Genes were considered adjacent to base J and/or H3.V if the gene, according to the *T*. *brucei* Lister 427 annotation, overlapped within 10,000 base pairs upstream or downstream of the modification. All J IP-seq and H3.V ChIP-seq data shown here are from previously published work [13, 14]. Fold changes for the heatmaps were computed as (RPKM_var_ + pseudocount) / (RPKM_wt_ + pseudocount), where pseudocount = 0.5. Once all fold changes were computed, any fold change value above five was set equal to five to improve visualization.

### Strand-specific RT-PCR analysis of read through transcription

Total RNA was isolated using the hot phenol method, as described previously [98]. To ensure complete removal of contaminating genomic DNA, purified RNA was treated with Turbo DNase, followed by phenol:chloroform extraction. RNA concentration was determined using a spectrophotometer. Strand specific RT-PCR was performed as previously described [99]. ThermoScript Reverse Transcriptase from Life Technologies was used for cDNA synthesis at 60–65°C. 1–2 μg of RNA were used to make cDNA using a reverse primer as described in the Figure legends. PCR was performed using GoTaq DNA Polymerase from Promega. A minus-RT control was used to ensure no contaminating genomic DNA was amplified. Primer sequences used in the analysis are available upon request.

### Reverse transcription quantitative PCR (RT qPCR)

Total RNA was obtained using Qiagen RNeasy kits according to manufacturer’s instructions. First-strand cDNA was synthesized from 1 μg of total RNA using an iScript cDNA synthesis kit (Bio-Rad Laboratories, Hercules, CA) per the manufacturer's instructions. Quantification of selected genes were performed on an iCycler with an iQ5 multicolor real-time PCR detection system (Bio-Rad Laboratories, Hercules, CA). Primer sequences used in the analysis are available upon request. The reaction mixture contained 5 pmol forward and reverse primer, 2x iQ SYBR green super mix (Bio-Rad Laboratories, Hercules, CA), and 2 μl of template cDNA. Standard curves were prepared for each gene using 5-fold dilutions of known quantity (100 ng/μl) of WT DNA. The quantities were calculated using iQ5 optical detection system software.

## Supporting Information

S1 Fig Regulation of siRNAs by H3.V. (A) Mapping of 23-26nt small RNAs to cSSR 11.9 in WT and *H3*.*V* KO. (B) Phasing of siRNAs mapping to a cSSR on chromosome 5 in WT cells. Position is indicated in kb. Colors indicate nucleotide: green, A; red, T; blue, C; and orange, G.
(TIFF)

S2 Fig Regulation of siRNAs by H3.V at other loci. Mapping of small RNAs to SLACS, INGI, CIR147 and IR3.
(TIFF)

S3 Fig Regulation of termination and gene expression by H3.V. (A-C) Localization of H3.V, J, ORFs, and mRNA-seq reads from wild type *T*. *brucei* are plotted for cSSR 9.2 (position 1110–1190 kb is shown). (D-F) Gene expression changes and termination defects are analyzed as described in Fig 3. P values were calculated using Student’s t test. *, p value ≤ 0.05; **, p value ≤ 0.01.
(TIFF)

S4 Fig Confirmation of mRNA-seq transcript changes in *T*. *brucei* by RT-qPCR. RT-qPCR was performed as described in Fig 3D. 5350: *Tb427tmp*.*160*.*5350*; 1960: *Tb427*.*07*.*1960*; 1565: *Tb427tmp*.*02*.*1565*; 1580: *Tb427tmp*.*02*.*1580*; 5384: *Tb427*.*02*.*5384*; and 6820: *Tb427*.*07*.*6820*. P values were calculated using Student’s t test. *, p value ≤ 0.05; **, p value ≤ 0.01.
(TIFF)

S5 Fig Regulation of termination and gene expression by H3.V. (A-C) Localization of H3.V, J, ORFs and mRNA-seq reads from wild type *T*. *brucei* are ploted for cSSR 10.5 (1120–1140). (D) mRNA-seq transcript fold changes of the genes indicated in the ORF map in B, as described in Fig 5E. White bars: Wild type; grey bars: Wild type+DMOG; dark grey bars: *H3*.*V* KO; black bars: *H3*.*V* KO+DMOG. The fold change in the wild type+DMOG condition for gene 4 is 12.2, with a standard deviation of 4.3 and p value of 0.03.
(TIFF)

S6 Fig Chromosome maps. Whole chromosomes (Chr. 1–11, *T*. *brucei* Lister 427 version 9.0 genome) and the localization of base J (blue), H3.V (red), and mRNA coding genes (black lines; top strand is indicated by a line in the top half of the panel, bottom strand by a line in the bottom half) are shown. Genes on the top strand are transcribed from left to right and those on the bottom strand are transcribed from right to left. Position along each chromosome is indicated in kilobases (KB) or megabases (MB). Bottom two panels: mRNAs found upregulated by at least 2-fold or more in the *H3*.*V* KO (top) and *H3*.*V* KO+DMOG (bottom) relative to WT are indicated by a green line. Only mRNAs with an RPKM≥1 and significantly differentially expressed relative to wild type, as determined by Cuffdiff, are included. Boxes indicate sites examined in more detail in other figures. Genes are listed in S1 and S4 Tables.
(PDF)

S7 Fig Enrichment of genes adjacent to H3.V and J following the loss of H3.V and/or J. (A) Genes were defined as adjacent to H3.V or J if located within 10 kb of an H3.V and J enriched region, respectively, and are indicated in grey. Genes not adjacent to H3.V or J are indicated in white [13, 14]. 2463 (27%) genes are adjacent to H3.V and 2837 (31%) are adjacent to J out of a total of 9266 annotated genes in the *T*. *brucei* genome. (B) 153 genes were upregulated in the absence of H3.V and/or J, 122 are adjacent to H3.V and 121 are adjacent to J (80%). 65 genes were downregulated in the absence of H3.V and/or J, 35 are adjacent to H3.V (54%) and 40 are adjacent to J (62%).
(TIFF)

S8 Fig Genomic context of genes downregulated in the *H3*.*V* KO. (A) The EP/PAG1 loci. Small arrow indicates the RNAP I transcription start site in the promoter region. Genes in bold are downregulated in the *H3*.*V* KO. Genes in blue are transcribed by RNAP II on the top strand and EP and PAG genes in red are transcribed by RNAP I. MARP: microtubule-associated repetitive protein; EP1-2: procyclin; PAG; procyclin associated gene; T: ‘T region’ encoding transcripts containing small ORFs of <240 bp; GU2: gene of unknown function. The Fig is drawn to scale. (B) Gene cluster on chromosome 6. Genes in bold are downregulated in the *H3*.*V* KO and identities are listed in S1 Table.
(TIFF)

S9 Fig RT-qPCR analysis of ES associated *ESAGs*. RT-qPCR analysis of the indicated *ESAGs* was performed as described in Fig 3D. P values were calculated using Student’s t test. *, p value ≤ 0.05; **, p value ≤ 0.01.
(TIFF)

S10 Fig Working model for H3.V regulating RNAP II transcription and mRNA and siRNA expression. O-linked glycosylation of DNA (base J) is indicated by black line and dot. Nucleosomes are indicated by circles where green represents canonical histones and red represents histone H3 variant and an additional histone variant found at termination sites in *T*. *brucei* [14], histone H4 variant (H4.V). In the *H3*.*V* KO the H3.V is replaced with a canonical H3, with no change in nucleosome structure or H4.V, since it is currently unclear what happens to the nucleosome upon the loss of H3.V. According to the model, (A) the loss of base J leads to read-through transcription (indicated by the thicker red arrow) at internal termination sites within the cluster that is once again attenuated once it reaches H3.V within the cSSR. The loss of H3.V leads to read-through transcription at the internal site and continues into the cSSR, thus allowing increased dual strand transcription and generation of siRNAs. (B) At regions without an internal termination site, loss of base J has no effect on dual strand transcription. But, as described above, the loss of H3.V leads to increased transcription at cSSRs and generation of siRNAs.
(TIFF)

S1 Table *T*. *brucei* gene expression changes following H3.V and/or J loss. mRNAs found up or downregulated by 2-fold or more by mRNA-seq are listed, along with available gene descriptions, RPKM, fold change, and an indication of whether the genes are located within 10kb of H3.V and/or J. P values determined by Cuffdiff are listed for each condition compared to WT. For upregulated mRNAs, only those with an RPKM≥1 upon the loss of H3.V and/or J are included. For downregulated mRNAs, only those with an RPKM≥1 in WT cells are included.
(XLSX)

S2 Table Small RNA-seq RPM and statistical significance at cSSRs. The average small RNA-seq RPM of triplicate libraries at cSSRs is listed. Chromosome number and the 5’ and 3’ position of regions quantified are included. Statistical significance was assessed using pairwise Fisher’s Exact test on both total reads and specifically 21-27bp reads. Yellow highlight indicates significance at a p value ≤ 0.05.
(XLSX)

S3 Table High-throughput sequencing information. Information about all sequencing experiments performed in this study is listed. Also indicates the figures in which the data are presented.
(XLSX)

S4 Table *T*. *brucei* upregulated genes following H3.V and/or J loss. Similar to S1 Table, but upregulated genes are organized according to their location within PTUs. Genes sharing the same number are located in the same PTU and genes with different numbers are located in different PTUs.
(XLSX)
