# Supplementary figures and images for: Histone H3 Variant Regulates RNA Polymerase II Transcription Termination and Dual Strand Transcription of siRNA Loci in Trypanosoma brucei
Source: PLoS Genet. 2016 Jan 21;12(1):e1005758. doi: 10.1371/journal.pgen.1005758 (PMC4721609; doi:10.1371/journal.pgen.1005758)

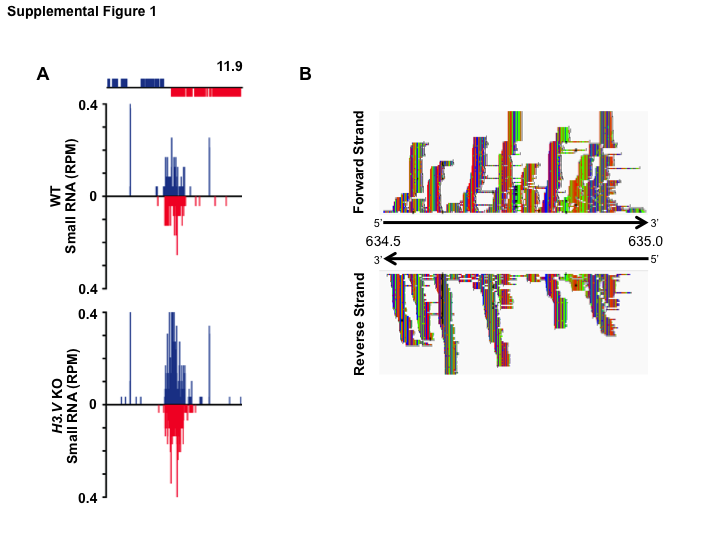

Supplement: S1 Fig — (A) Mapping of 23-26nt small RNAs to cSSR 11.9 in WT and H3.V KO. (B) Phasing of siRNAs mapping to a cSSR on chromosome 5 in WT cells. Position is indicated in kb. Colors indicate nucleotide: green, A; red, T; blue, C; and orange, G. (TIFF) [file pgen.1005758.s001.tiff]

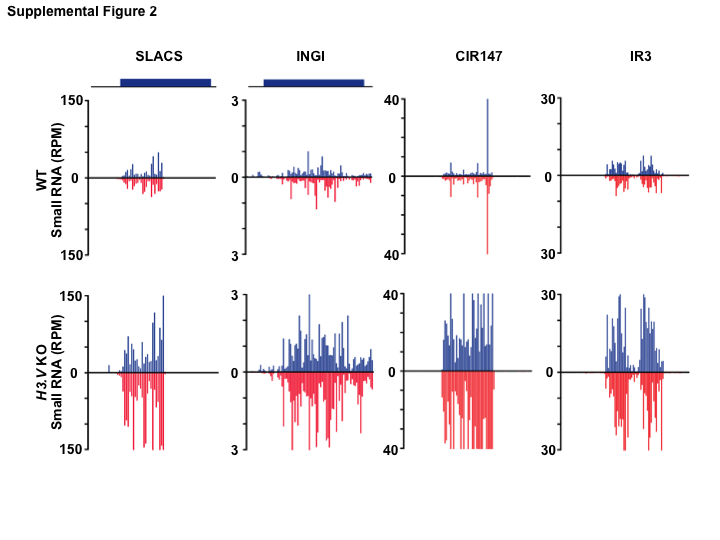

Supplement: S2 Fig — Mapping of small RNAs to SLACS, INGI, CIR147 and IR3. (TIFF) [file pgen.1005758.s002.tiff]

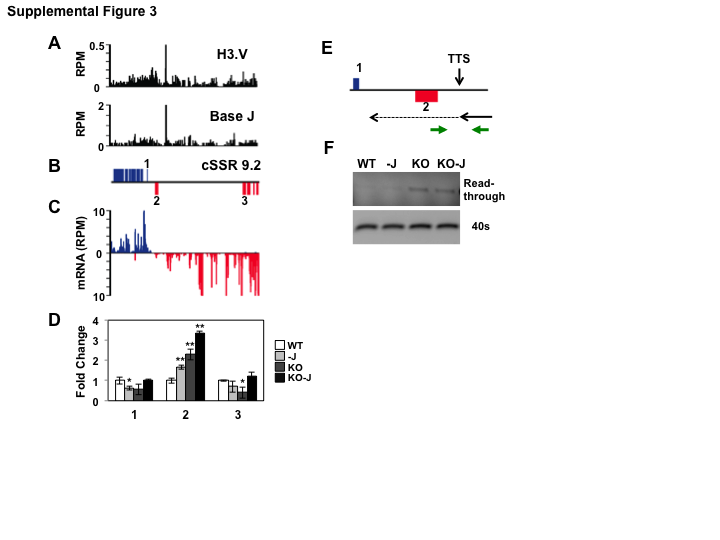

Supplement: S3 Fig — (A-C) Localization of H3.V, J, ORFs, and mRNA-seq reads from wild type T. brucei are plotted for cSSR 9.2 (position 1110–1190 kb is shown). (D-F) Gene expression changes and termination defects are analyzed as described in Fig 3. P values were calculated using Student’s t test. *, p value ≤ 0.05; **, p value ≤ 0.01. (TIFF) [file pgen.1005758.s003.tiff]

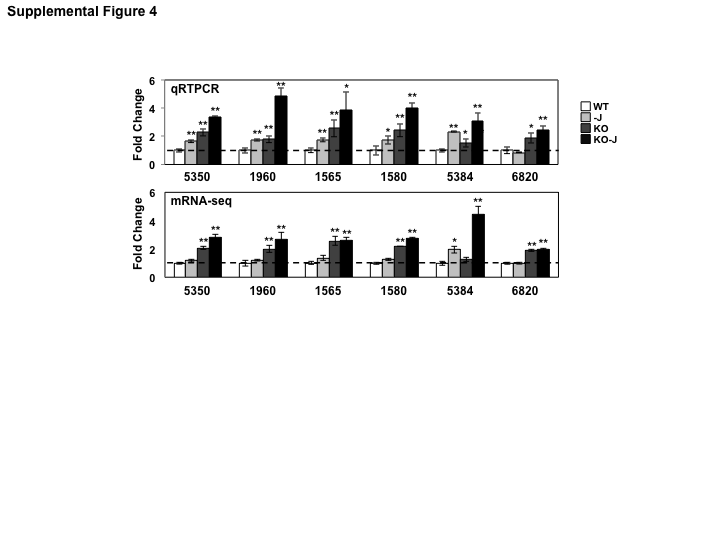

Supplement: S4 Fig — RT-qPCR was performed as described in Fig 3D. 5350: Tb427tmp.160.5350; 1960: Tb427.07.1960; 1565: Tb427tmp.02.1565; 1580: Tb427tmp.02.1580; 5384: Tb427.02.5384; and 6820: Tb427.07.6820. P values were calculated using Student’s t test. *, p value ≤ 0.05; **, p value ≤ 0.01. (TIFF) [file pgen.1005758.s004.tiff]

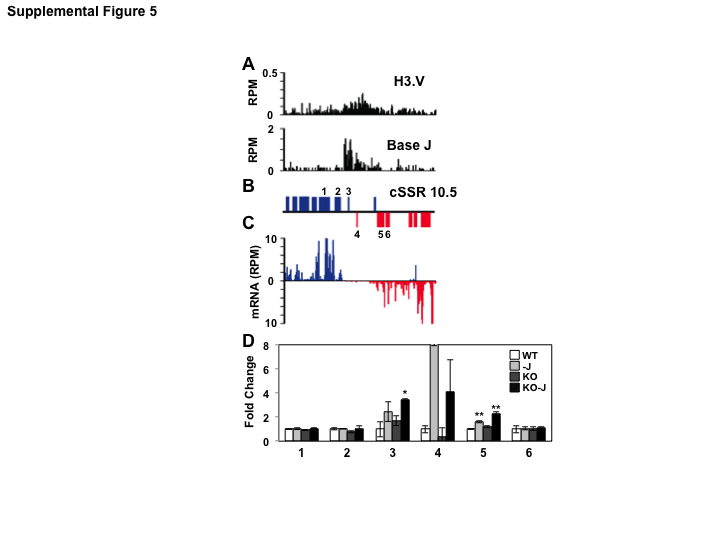

Supplement: S5 Fig — (A-C) Localization of H3.V, J, ORFs and mRNA-seq reads from wild type T. brucei are ploted for cSSR 10.5 (1120–1140). (D) mRNA-seq transcript fold changes of the genes indicated in the ORF map in B, as described in Fig 5E. White bars: Wild type; grey bars: Wild type+DMOG; dark grey bars: H3.V KO; black bars: H3.V KO+DMOG. The fold change in the wild type+DMOG condition for gene 4 is 12.2, with a standard deviation of 4.3 and p value of 0.03. (TIFF) [file pgen.1005758.s005.tiff]

# Chr. 1

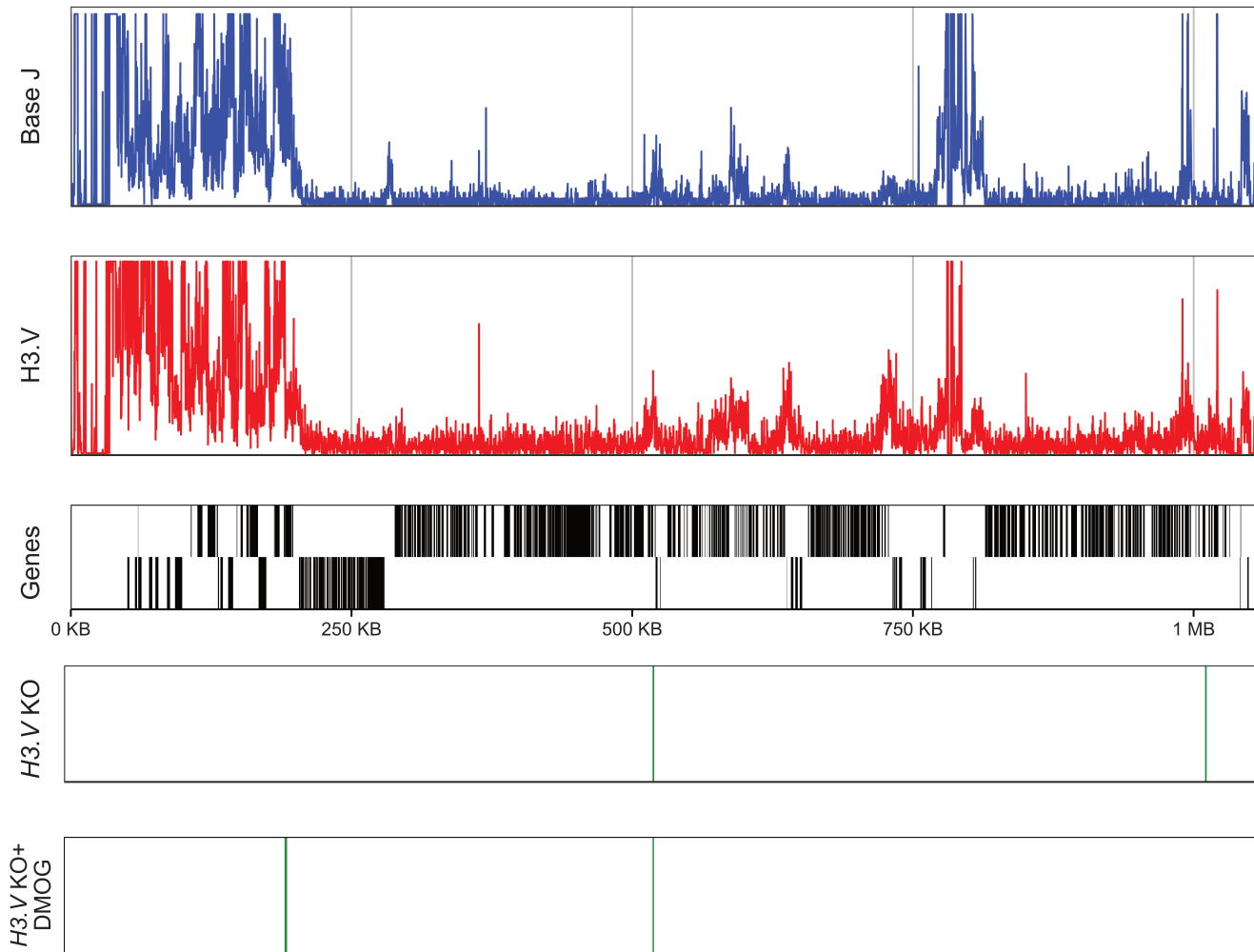

# Chr. 2

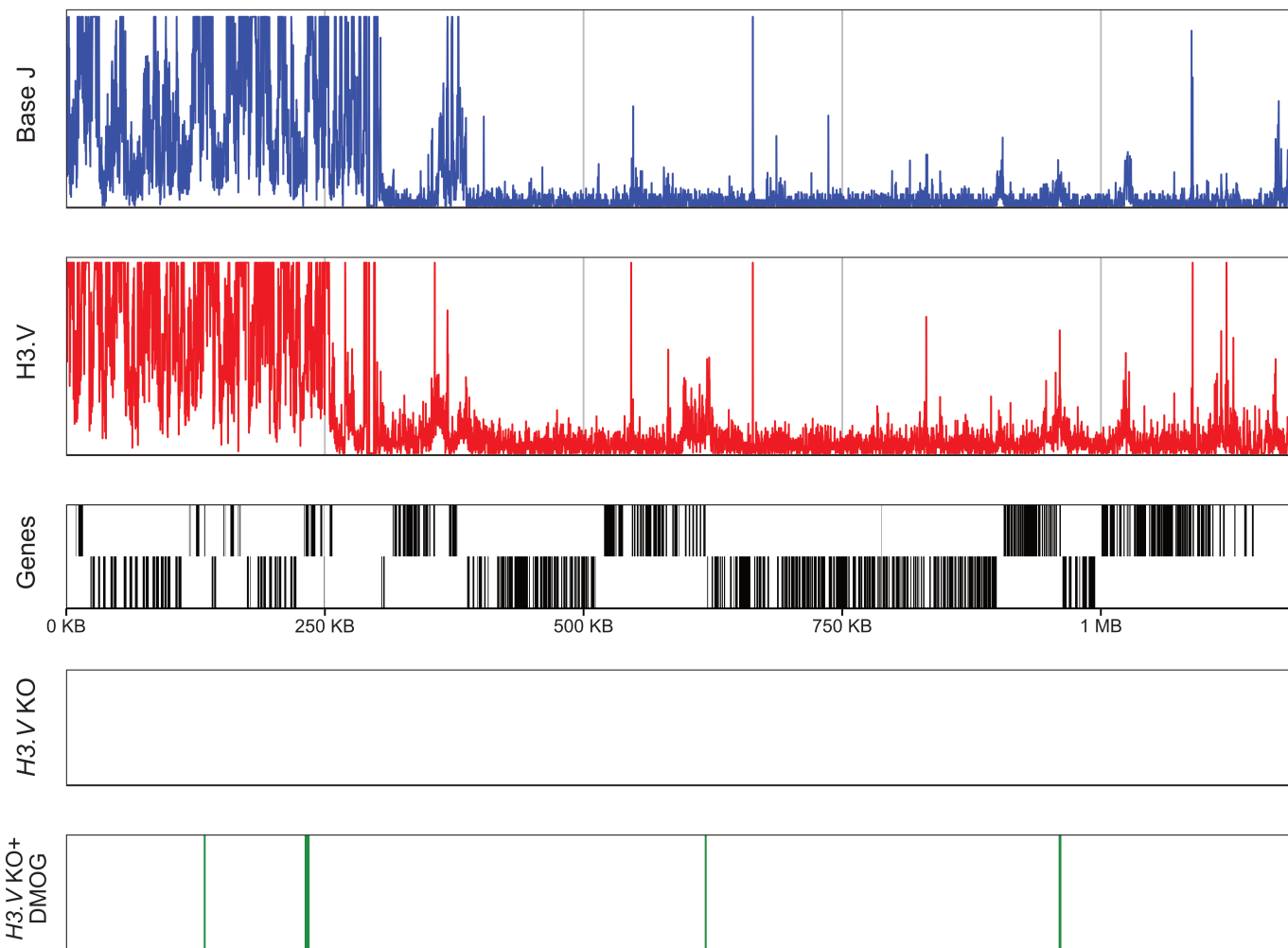

# Chr. 3

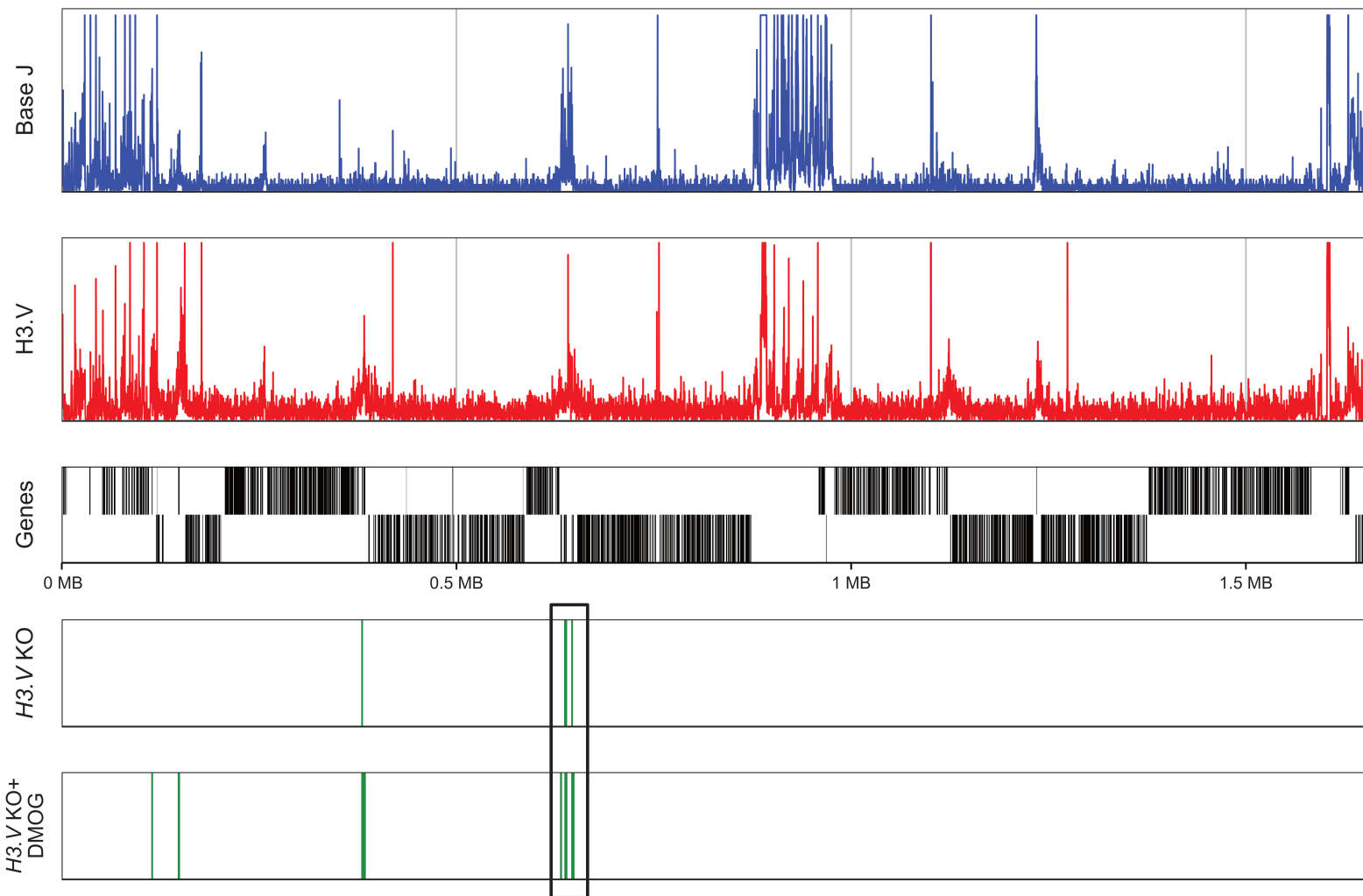

# Chr. 4

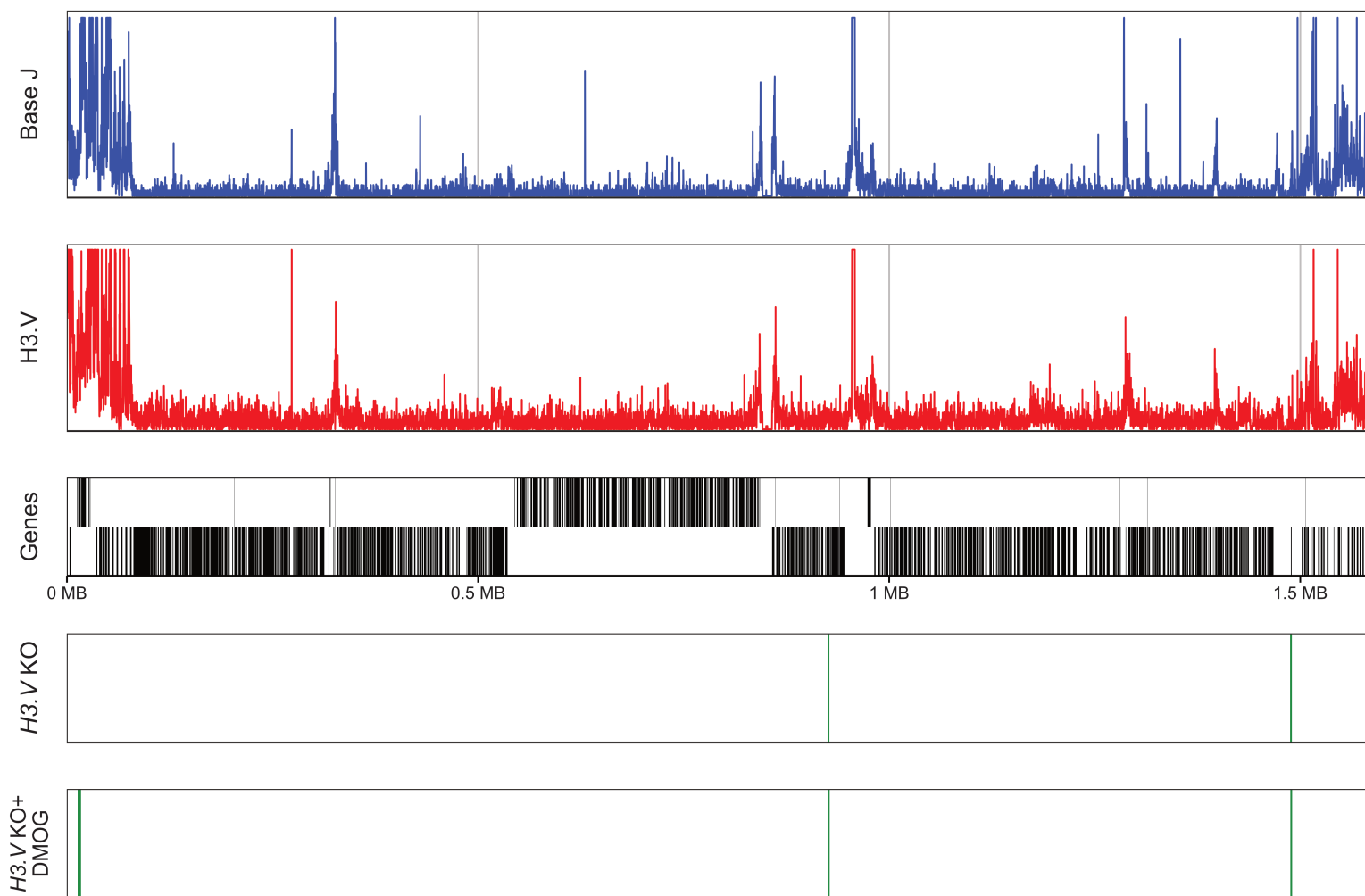

# Chr. 5

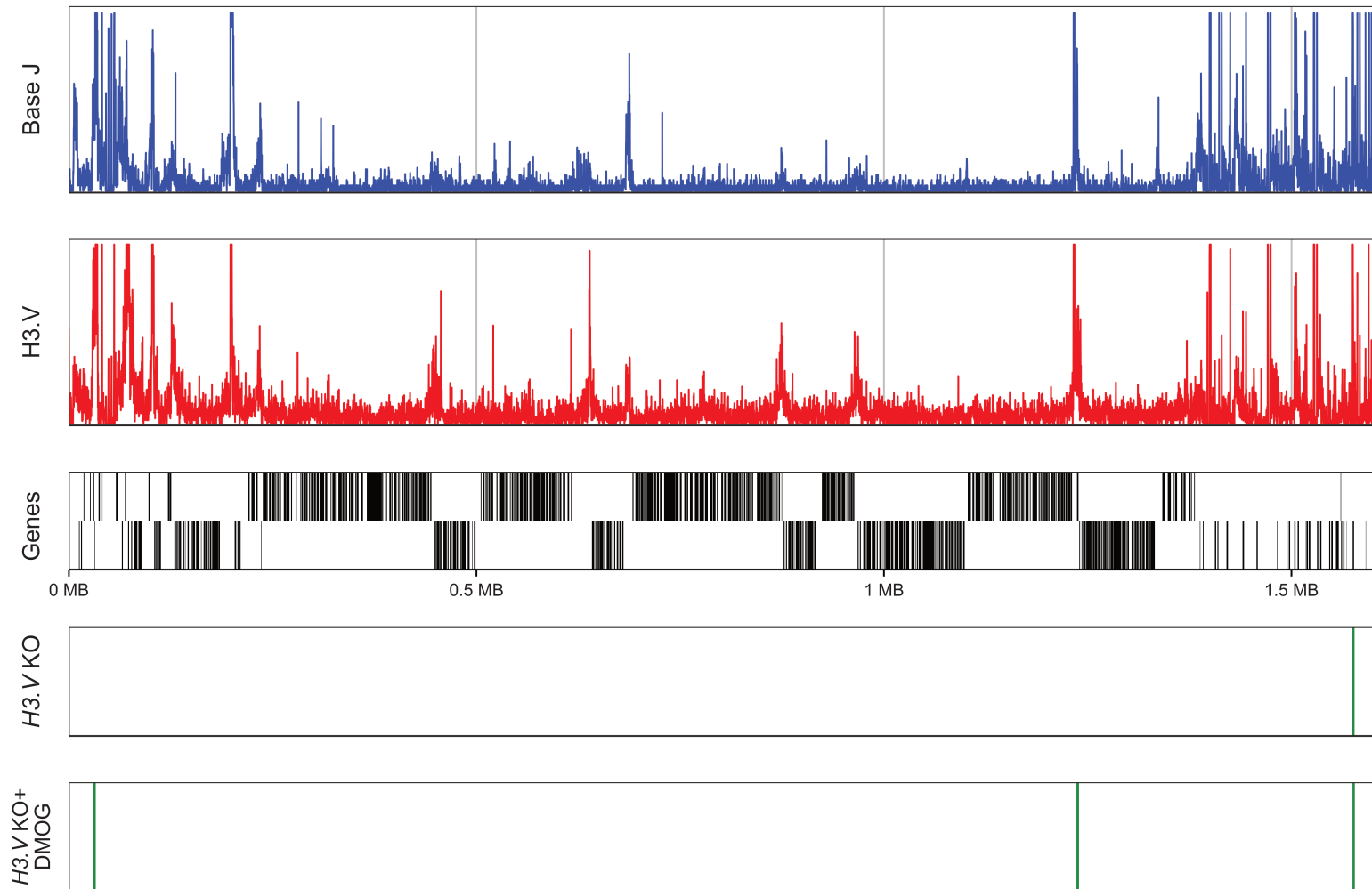

# Chr. 6

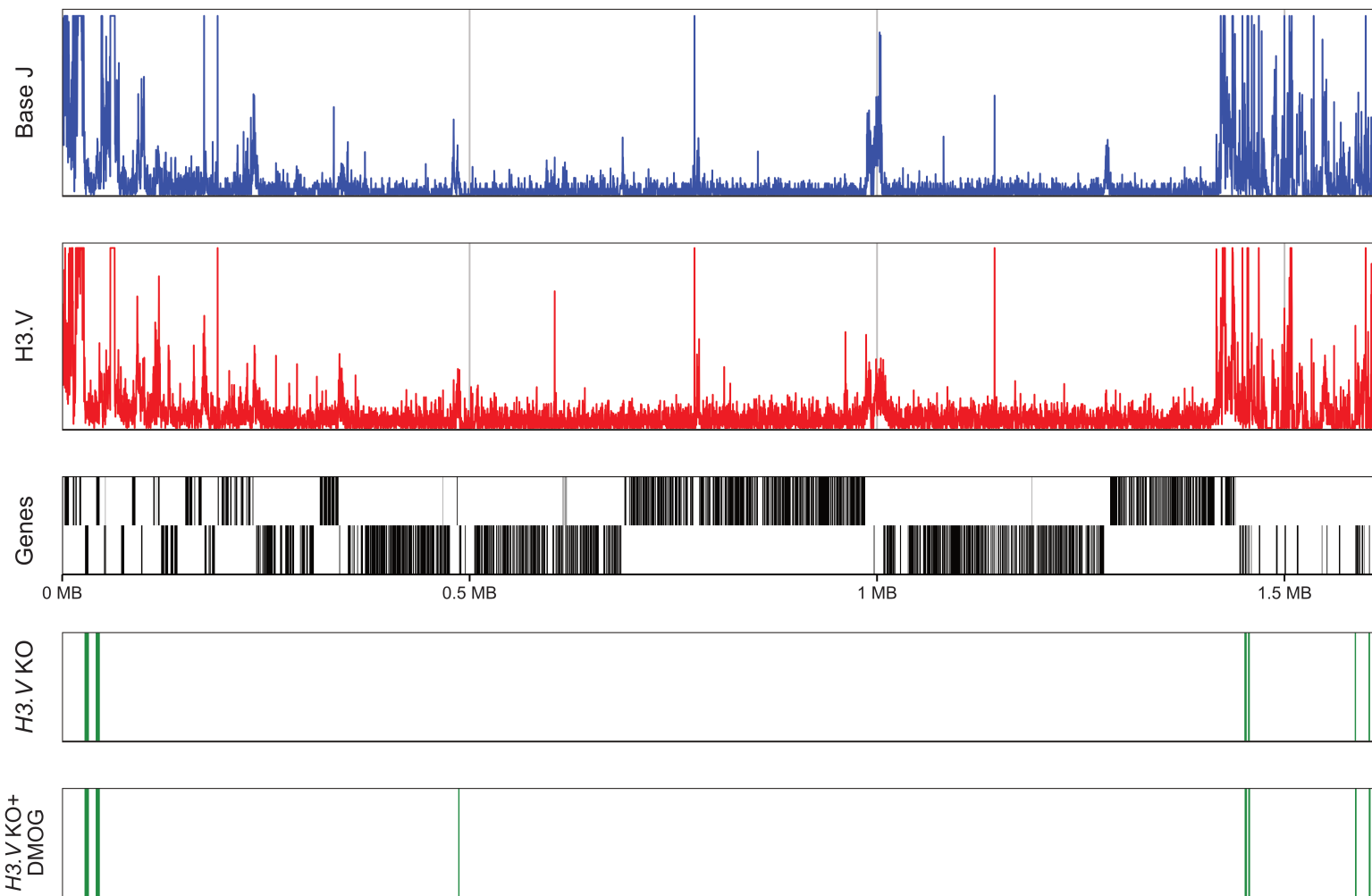

# Chr. 7

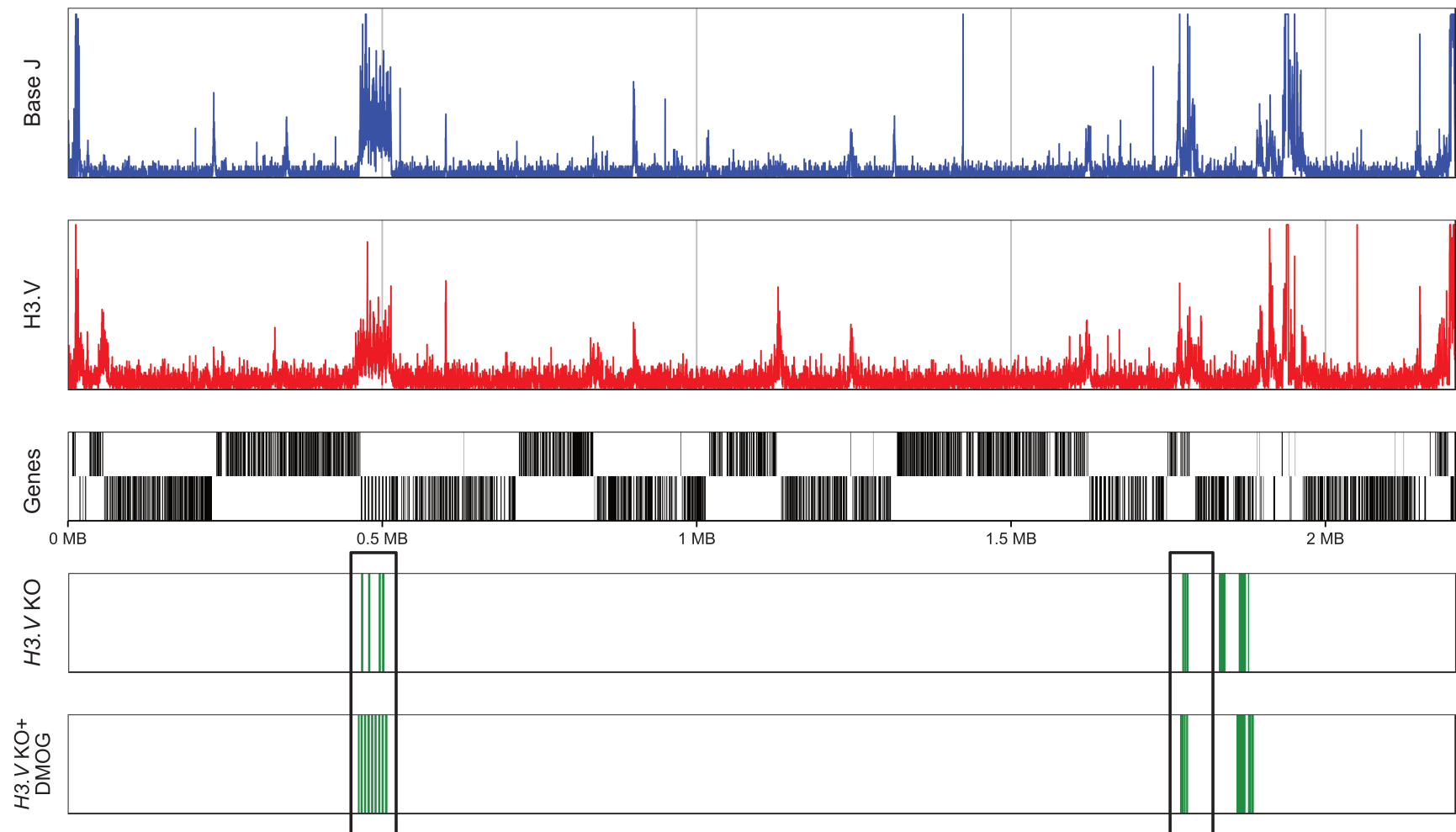

# Chr. 8

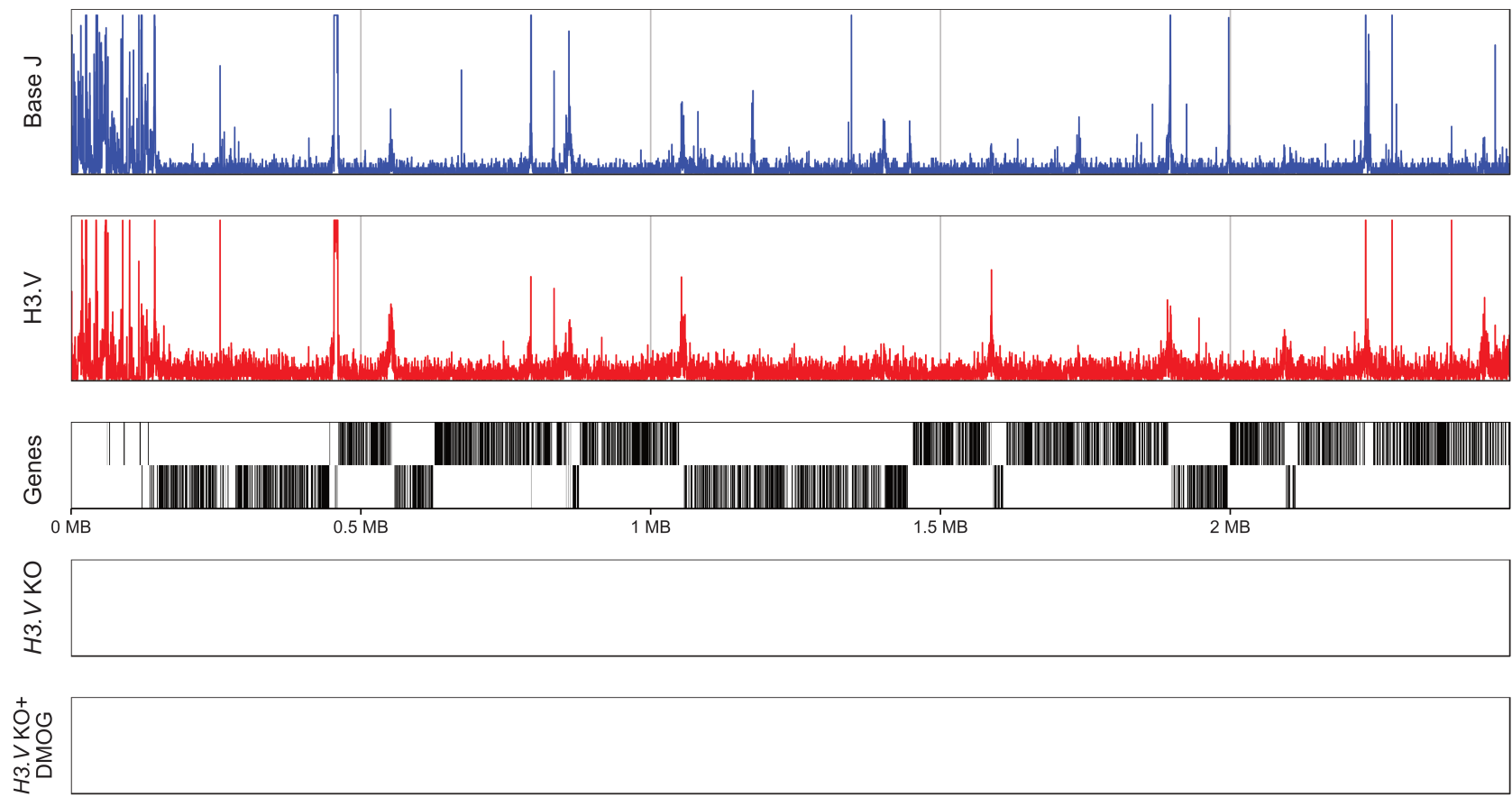

# Chr. 9

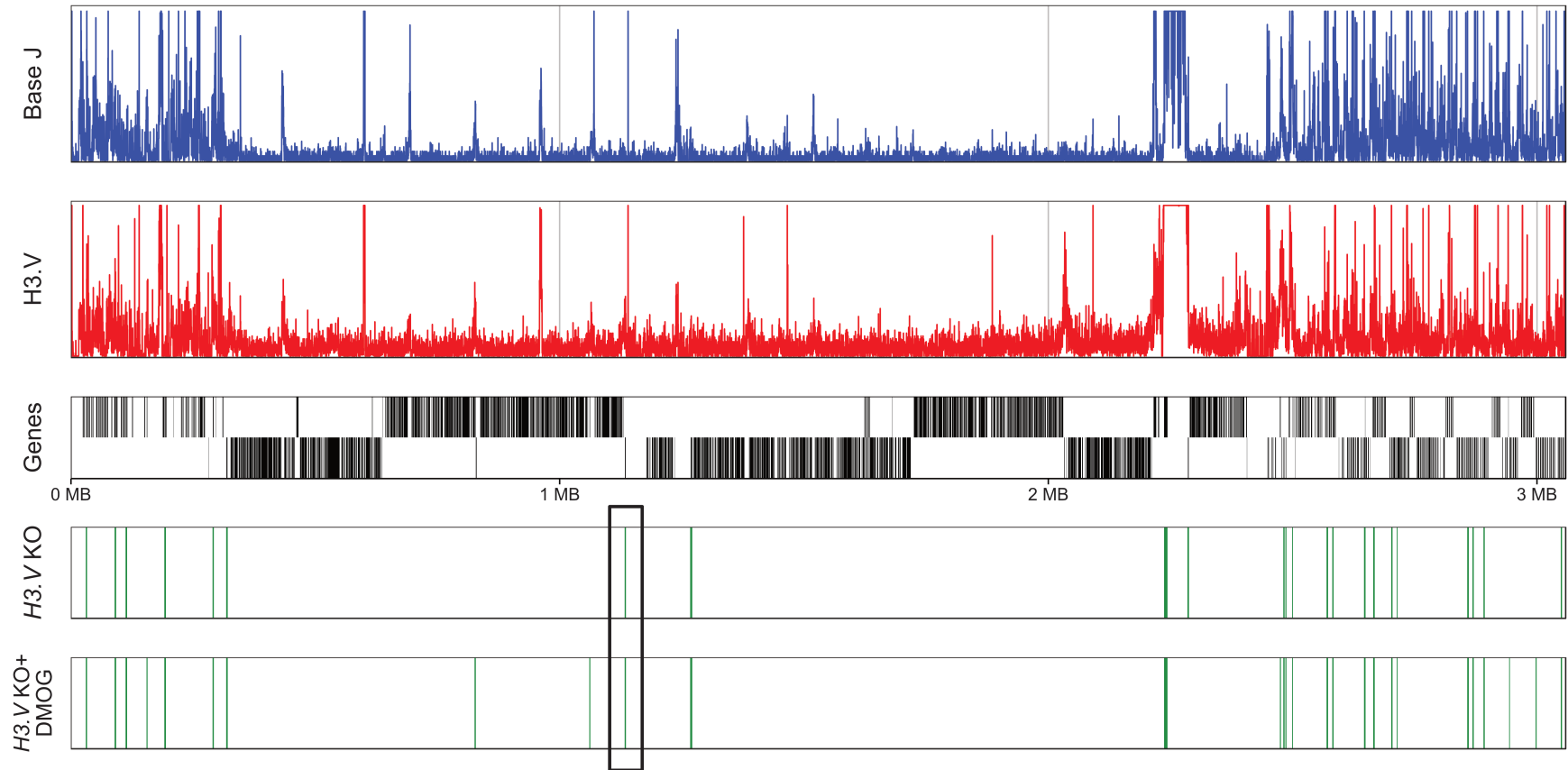

Chr. 10

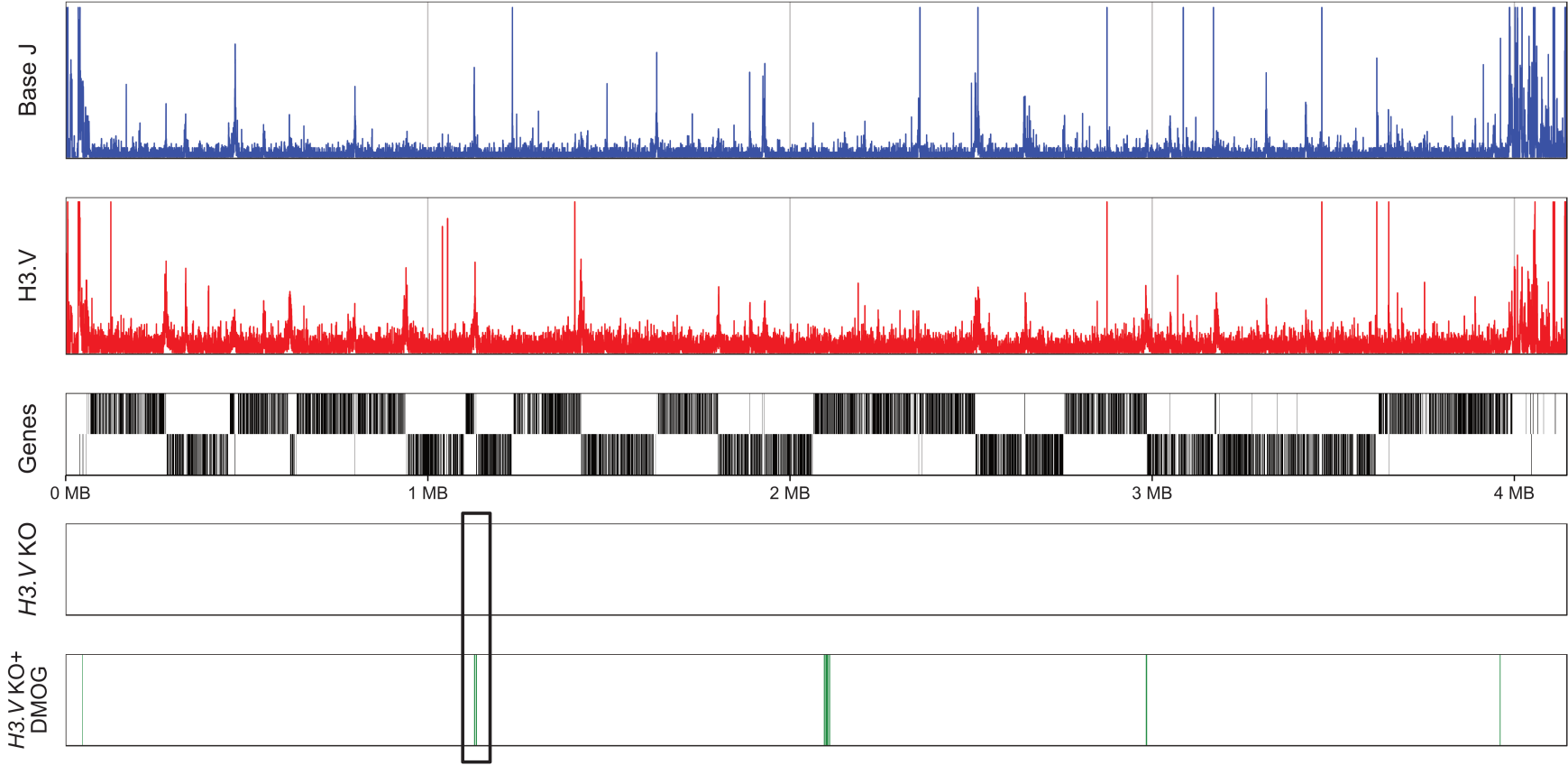

## Chr. 11

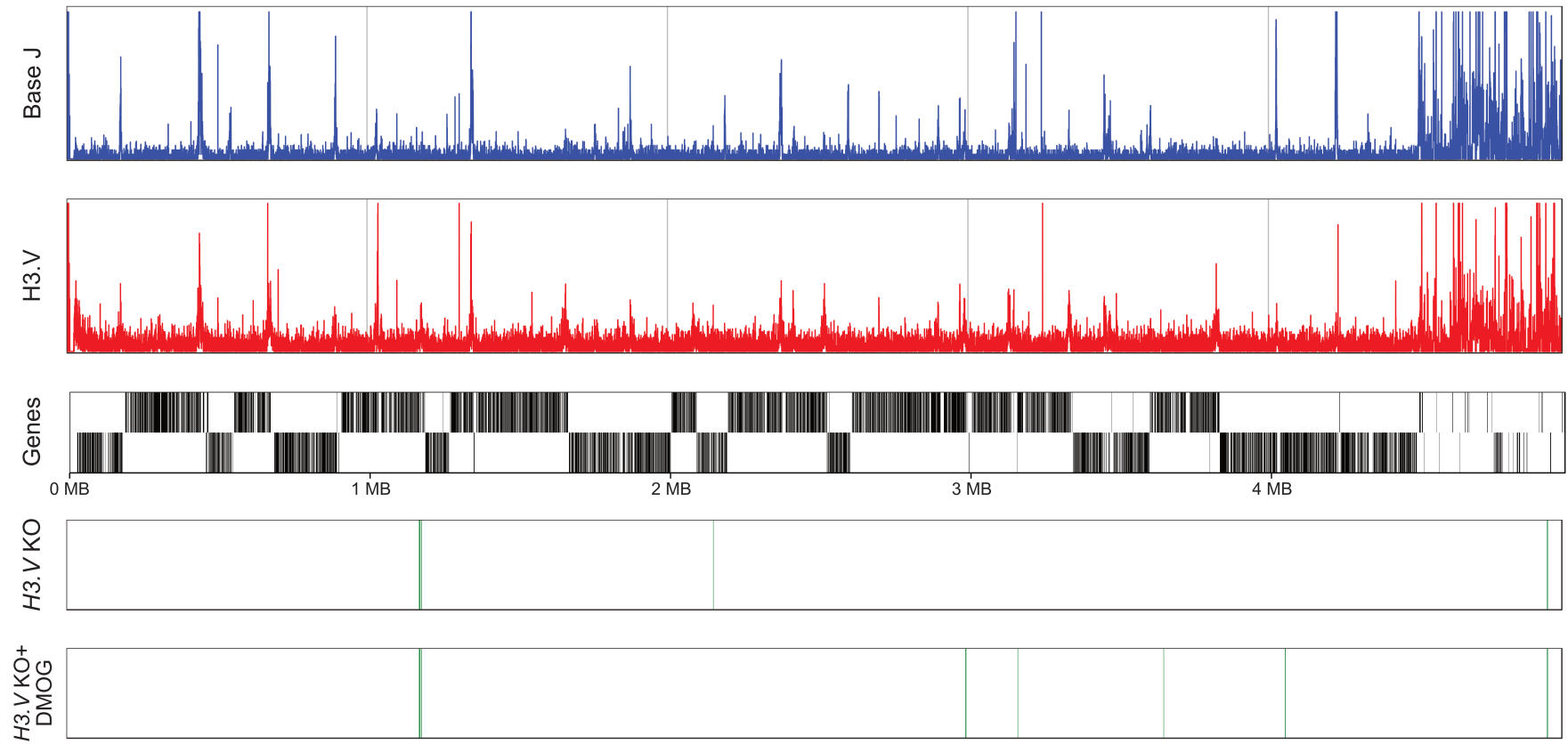

Supplement: S6 Fig — Whole chromosomes (Chr. 1–11, T. brucei Lister 427 version 9.0 genome) and the localization of base J (blue), H3.V (red), and mRNA coding genes (black lines; top strand is indicated by a line in the top half of the panel, bottom strand by a line in the bottom half) are shown. Genes on the top strand are transcribed from left to right and those on the bottom strand are transcribed from right to left. Position along each chromosome is indicated in kilobases (KB) or megabases (MB). Bottom two panels: mRNAs found upregulated by at least 2-fold or more in the H3.V KO (top) and H3.V KO+DMOG (bottom) relative to WT are indicated by a green line. Only mRNAs with an RPKM≥1 and significantly differentially expressed relative to wild type, as determined by Cuffdiff, are included. Boxes indicate sites examined in more detail in other figures. Genes are listed in S1 and S4 Tables. (PDF) [file pgen.1005758.s006.pdf]

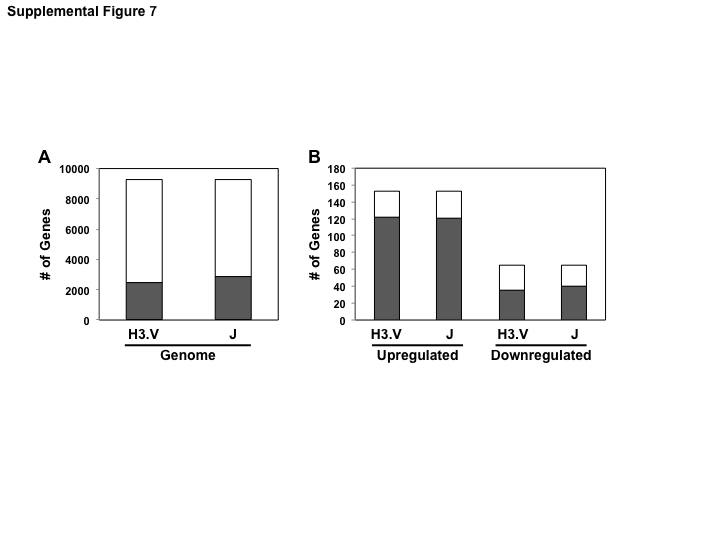

Supplement: S7 Fig — (A) Genes were defined as adjacent to H3.V or J if located within 10 kb of an H3.V and J enriched region, respectively, and are indicated in grey. Genes not adjacent to H3.V or J are indicated in white [13, 14]. 2463 (27%) genes are adjacent to H3.V and 2837 (31%) are adjacent to J out of a total of 9266 annotated genes in the T. brucei genome. (B) 153 genes were upregulated in the absence of H3.V and/or J, 122 are adjacent to H3.V and 121 are adjacent to J (80%). 65 genes were downregulated in the absence of H3.V and/or J, 35 are adjacent to H3.V (54%) and 40 are adjacent to J (62%). (TIFF) [file pgen.1005758.s007.tiff]

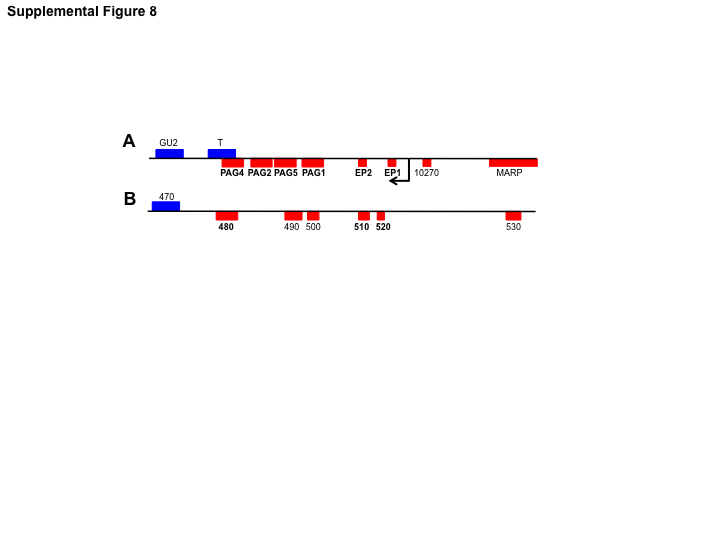

Supplement: S8 Fig — (A) The EP/PAG1 loci. Small arrow indicates the RNAP I transcription start site in the promoter region. Genes in bold are downregulated in the H3.V KO. Genes in blue are transcribed by RNAP II on the top strand and EP and PAG genes in red are transcribed by RNAP I. MARP: microtubule-associated repetitive protein; EP1-2: procyclin; PAG; procyclin associated gene; T: ‘T region’ encoding transcripts containing small ORFs of <240 bp; GU2: gene of unknown function. The Fig is drawn to scale. (B) Gene cluster on chromosome 6. Genes in bold are downregulated in the H3.V KO and identities are listed in S1 Table. (TIFF) [file pgen.1005758.s008.tiff]

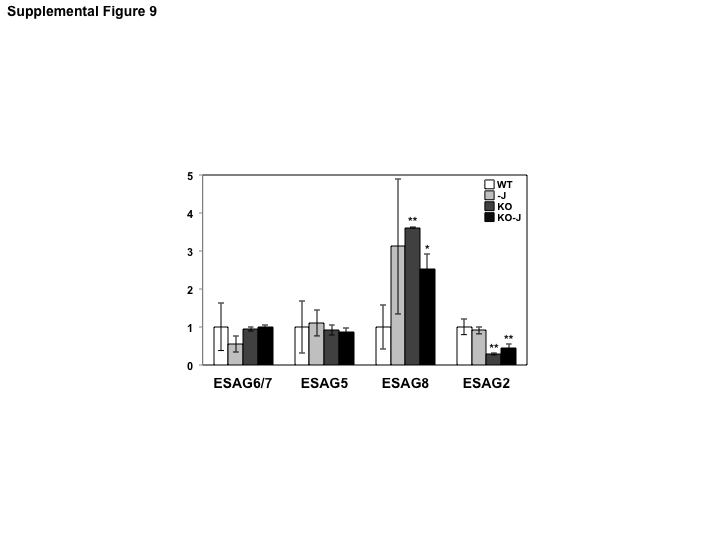

Supplement: S9 Fig — RT-qPCR analysis of the indicated ESAGs was performed as described in Fig 3D. P values were calculated using Student’s t test. *, p value ≤ 0.05; **, p value ≤ 0.01. (TIFF) [file pgen.1005758.s009.tiff]

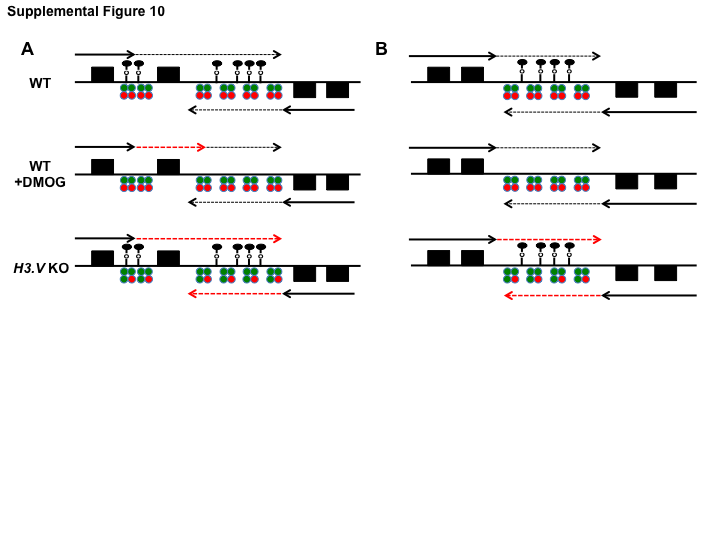

Supplement: S10 Fig — O-linked glycosylation of DNA (base J) is indicated by black line and dot. Nucleosomes are indicated by circles where green represents canonical histones and red represents histone H3 variant and an additional histone variant found at termination sites in T. brucei [14], histone H4 variant (H4.V). In the H3.V KO the H3.V is replaced with a canonical H3, with no change in nucleosome structure or H4.V, since it is currently unclear what happens to the nucleosome upon the loss of H3.V. According to the model, (A) the loss of base J leads to read-through transcription (indicated by the thicker red arrow) at internal termination sites within the cluster that is once again attenuated once it reaches H3.V within the cSSR. The loss of H3.V leads to read-through transcription at the internal site and continues into the cSSR, thus allowing increased dual strand transcription and generation of siRNAs. (B) At regions without an internal termination site, loss of base J has no effect on dual strand transcription. But, as described above, the loss of H3.V leads to increased transcription at cSSRs and generation of siRNAs. (TIFF) [file pgen.1005758.s010.tiff]
